# Sleep disturbance as a moderator of the association between physical activity and later pain onset among American adults aged 50 and over: evidence from the Health and Retirement Study

**DOI:** 10.1136/bmjopen-2019-036219

**Published:** 2020-06-07

**Authors:** Daniel Whibley, Heidi M Guyer, Leslie M Swanson, Tiffany J Braley, Anna L Kratz, Galit Levi Dunietz

**Affiliations:** 1 Epidemiology Group, School of Medicine, Medical Sciences and Nutrition, University of Aberdeen, Aberdeen, Scotland, United Kingdom; 2 Department of Physical Medicine and Rehabilitation, University of Michigan, Ann Arbor, Michigan, United States; 3 Department of Anesthesiology, Chronic Pain and Fatigue Research Center, University of Michigan, Ann Arbor, Michigan, United States; 4 Survey Research Center, Institute for Social Research, University of Michigan, Ann Arbor, Michigan, United States; 5 RTI International, North Carolina, United States; 6 Department of Psychiatry, University of Michigan, Ann Arbor, Michigan, United States; 7 Department of Neurology, Division of Multiple Sclerosis and Neuroimmunology, University of Michigan, Ann Arbor, Michigan, United States; 8 Department of Neurology, Division of Sleep Medicine, University of Michigan, Ann Arbor, Michigan, United States

**Keywords:** physical activity, sleep disturbance, pain, older adults

## Abstract

**Objective:**

To examine whether sleep disturbance modifies the association between physical activity and incident pain.

**Design:**

Prospective population-based study.

**Setting:**

Health and Retirement Study.

**Participants:**

American adults aged ≥50 years who reported no troublesome pain in 2014 were re-assessed for pain in 2016. Of 9828 eligible baseline respondents, 8036 (82%) had complete follow-up data for adjusted analyses (weighted analysis population N=42 407 222).

**Exposures:**

Physical activity was assessed via interview with questions about time spent in moderate and vigorous physical activity. Sleep disturbance, assessed using a modified form of the Jenkins Sleep Scale, was examined as a potential moderator.

**Main outcome measure:**

Troublesome pain.

**Results:**

In weighted analyses, 37.9% of the 2014 baseline pain-free sample participated in moderate or vigorous physical activity once a week or less, with an overall mean Physical Activity Index Score of 9.0 (SE=0.12). 18.6% went on to report troublesome pain in 2016. Each one-point higher on the Physical Activity Index Score was associated with a reduced odds ratio (OR) of incident pain for those who endorsed sleep disturbance never/rarely (OR=0.97, 95% CI 0.94 to 0.99), but not for those who endorsed sleep disturbance sometimes (OR=0.99, 95% CI 0.97 to 1.01) or most of the time (OR=1.01, 95% CI 0.99 to 1.03). The analysis of possible interaction demonstrated that frequency of sleep disturbance moderated the physical activity and incident pain association (Wald test: p=0.02).

**Conclusions:**

The beneficial association of physical activity on reduced likelihood of later pain was only observed in persons who endorsed low levels of sleep disturbance.

**Strengths and limitations of this study**

- This study uses data representative of the US population aged 50 years and over to provide evidence for the moderating effect of sleep disturbance on the association between the level of physical activity and later pain onset.
- Strengths of the study include its complex survey design, which supports population-level inference, and the low level of missing data.
- Limitations of the study include the self-reported nature of the variables under study, and the lack of available information on the presence of specific sleep disorders.

## Introduction

Pain is a pre-eminent public health issue,^1^ affecting approximately 20% of the global adult population.^2^ Its reach is pervasive, impacting on daily functioning,^3^ mental health^4^ and quality of life.^5^ The societal burden of pain is also vast, with annual economic costs, including those associated with healthcare and social services, exceeding $560 billion in the USA alone.^6^ Half of the older adult population in the USA is estimated to experience troublesome pain.^7^ With an increasingly older population^8^ and observed associations between pain onset and a reduction in healthy ageing,^9^ discovery of strategies to prevent or reduce the proliferation of pain in older adults to support healthy ageing is imperative. The identification of modifiable and protective lifestyle factors that contribute to pain is key to guide population-level efforts to achieve this aim.

One such modifiable factor, and an important determinant of healthy ageing, is physical activity.^10^ Physical activity is associated with lower levels of functional disability^11^ and morbidity,^12^ better mental health^13–15^ and a reduced likelihood of pain reporting.^16 17^ Accordingly, physical activity is a principal health behaviour included in the WHO’s 2015 World Report on Ageing and Health.^18^

Like low levels of physical activity, poor sleep quality and short sleep duration are associated with more functional disability,^19^ a wide range of diseases,^20–23^ poor mental health^24^ and pain reporting.^25–28^ Poor sleep quality and lower sleep efficiency are also associated with lower levels of physical activity and a decreased likelihood of uptake or maintenance of routine exercise.^29^ Conversely, higher levels of physical activity are associated with improvements in subjectively and objectively measured sleep.^30 31^ Despite this, advice about sleep behaviour is rarely included in physical activity guidance for older adults. A possible reason for this may be a tacit societal agreement that parameters of sleep (eg, duration, timing and continuity) inevitably decline with age. However, although changes in sleep duration, timing and continuity are observed as humans age, most changes occur during the transition from young to middle adulthood.^32^ Indeed, the National Sleep Foundation recommends that older adults sleep 7–8 hours per night (the recommendation for adults aged 18–64 is 7–9 hours).^33^

This study is predicated on the hypothesis that healthy sleep contributes to healthy ageing, and that sleep is an essential component, not only in its own right, but also as a condition within which the benefits of physical activity can be optimally reaped. To investigate the joint impact of sleep and physical activity on health, we examined whether sleep disturbance modified the association between the level of physical activity and likelihood of incident troublesome pain 2 years later in a nationally representative sample of older adults in the USA.

## Methods

### Study design

We used data from the Health and Retirement Study (HRS), a prospective survey of US adults aged 50 years and older conducted by the University of Michigan. Household face-to-face or telephone interviews are conducted with participants on a biannual basis across the contiguous USA.^34^ Data collection is conducted by trained interviewers using laptop computers. The complex survey design of HRS incorporates multistage area probability sampling, stratification, clustering and oversampling of adults of African American and Hispanic race/ethnicity. This allows HRS to provide a nationally representative sample that, with appropriate design-based analyses, can be used to make population-level inferences. A dataset comprising two HRS waves (2014 and 2016) was compiled and used to investigate relationships between physical activity, sleep disturbance and incident pain (those with no pain in 2014 followed forward for pain status in 2016). The HRS is sponsored by the National Institute on Aging.

## Measures

### Pain

The outcome was incident troublesome pain, determined by a ‘yes’ response to the question, ‘Are you often troubled with pain?’, in 2016. This item has previously been shown to be comparable to pain that at least moderately interferes with daily life.^35^

#### Physical activity

The WHO recommends that adults aged ≥65 years participate in 150 min of moderate physical activity or 75 min of vigorous physical activity each week, or an equivalent combination of both.^36^ The physical health section of HRS surveys includes questions about current levels of moderate and vigorous physical activity (moderate: ‘how often do you take part in sports or activities that are moderately energetic, such as gardening, cleaning the car, walking at a moderate pace, dancing, floor or stretching exercises?’; vigorous: ‘How often do you take part in sports or activities that are vigorous, such as running or jogging, swimming, cycling, aerobics or gym workout, tennis, or digging with a spade or shovel?’). Response options available for both questions are hardly ever or never, one to three times a month, once a week or more than once a week. Responses were used to create a Physical Activity Index Score, calculated following a previously used method.^37 38^ Moderate activity responses were coded as: 0=hardly ever, 1=one to three times a month, 3=once a week and 6=more than once a week. Vigorous activity responses were coded as: 0=hardly ever, 2=one to three times a month, 6=once a week and 12=more than once a week. A total Physical Activity Index Score was calculated by summing scored responses to these two questions (possible range: 0–18). To provide context for Physical Activity Index Scores, a binary variable was also created, guided by WHO’s physical activity recommendations. Respondents were categorised as participating in moderate or vigorous activity more than once a week, or not. According to this approach, Physical Activity Index Scores lower than 6 were indicative of physical inactivity; scores greater than 9 suggested meeting or exceeding WHO’s physical activity recommendations.

### Sleep disturbance

Sleep disturbances assessed at baseline (2014) were investigated as a potential moderator of the physical activity (2014) and later pain reporting (2016) association. Four questions about sleep disturbance type and frequency, adapted from the Jenkins Sleep Scale,^39^ were included in the physical health section of HRS: ‘How often do you have trouble falling asleep?’ ‘How often do you have trouble with waking up during the night?’ ‘How often do you have trouble with waking up too early and not being able to fall asleep again?’ ‘How often do you feel really rested when you wake up in the morning?’ Response options were: rarely or never; sometimes; or most of the time (reverse coded for the question about feeling rested on awakening to ensure consistency with other questions). Respondents were categorised as never or rarely experiencing sleep disturbance if they answered ‘rarely or never’ to all questions; sometimes, if they answered ‘sometimes’ but not ‘most of the time’ to any of these questions, and most of the time if they answered ‘most of the time’ to any of these questions.

### Demographic and health characteristics

Age, gender, race/ethnicity and number of years of school education were included as demographic covariates. Five health-related covariates were also adjusted for: body mass index (BMI), self-reported history of depression (‘Has a doctor ever told you that you have had problems with depression?’), major disease (self-reported history of cancer (any kind except skin), lung disease, heart condition or stroke), diabetes and arthritis.

## Statistical analysis

Analyses were conducted using Stata V.15.1 (StataCorp LLC, College Station, Texas, USA). HRS sample weights were used to correct for unequal probability of selection that may have arisen as a result of the multistage sampling design. Demographic and health-related characteristics were summarised (mean and SE). To investigate whether sleep disturbance reported in 2014 moderated the relationship between level of physical activity at this time and incident pain in 2016, logistic regression was performed with a Physical Activity Index Score–sleep disturbance status interaction term. As the research question focused on those with no troublesome pain at baseline, subpopulation estimation was used to compute point and variance estimates.^40^ This ensured that those reporting no pain at baseline contributed data to the calculation of effect estimates while those with and without pain at baseline contributed data to calculation of SEs. In the presence of significant interaction (p<0.05), predicted probabilities of reporting pain at follow-up were calculated and plotted according to Physical Activity Index Score and sleep disturbance status. Models were adjusted for age, gender, race/ethnicity, number of years of school education, BMI, self-reported history of depression, major diseases (self-reported history of cancer (any kind except skin), lung disease, heart condition or stroke), diabetes and arthritis.

### Patient involvement

Patients were not involved in this study and there are no plans to disseminate the findings to HRS participants.

## Results

### Descriptive analysis

Of 16 384 HRS respondents, 16 330 (99.7%) answered the question about troublesome pain in 2014. Of these, 9828 (60.2%) reported not being troubled by pain. Baseline demographic and health-related characteristics of this pain-free sample are presented in table 1. After applying sample weights, 51.5% were female, and the mean age was 66.9.

**Table 1:** Demographic and health characteristics of Health and Retirement Study respondents not troubled by pain in 2014

	Unweighted frequency (N)*	Weighted proportion or mean (SE)*
Gender		
Female	5346	51.5% (0.005)
Male	4482	48.5% (0.005)
Age (mean and SE)	9828	66.9 (0.3)
BMI		
Underweight/normal weight	2816	31.2% (0.006)
Overweight	3426	38.9% (0.007)
Obese	1820	20.1% (0.006)
Obese, BMI ≥35	920	9.9% (0.004)
Missing data	846 (8.6%)	
Race/ethnicity		
White	6371	78.7% (0.01)
Black	1837	9.6% (0.006)
Hispanic	1301	8.4% (0.01)
Other	319	3.4% (0.003)
Years of school (mean and SE)	9792	13.5 (0.08)
Missing data	36 (0.4%)	
Marital status in 2014		
Married	5759	62.6% (0.007)
Separated or divorced	1644	16.2% (0.005)
Widowed	1943	14.4% (0.004)
Never married	481	6.8% (0.004)
Missing data	1 (0.01%)	
Self-rated health		
Excellent	1131	14.0% (0.005)
Very good	3611	40.6% (0.008)
Good	3255	30.7% (0.007)
Fair	1499	12.0% (0.006)
Poor	323	2.5% (0.002)
Do not know/refused	9 (0.09%)	
History of depression		
Yes	1554	16.8% (0.005)
No	8203	83.2% (0.005)
Missing data	71 (0.7%)	
History of major disease†		
Yes	3766	35.5% (0.008)
No	5960	64.5% (0.008)
Missing data	102 (1.0%)	
Alzheimer’s disease or dementia		
Yes	274	2.0% (0.001)
No	9510	98.0% (0.001)
Missing data	44 (0.5%)	
Arthritis		
Yes	4653	43.8% (0.008)
No	5175	56.2% (0.008)
Diabetes		
Yes	2273	19.9% (0.005)
No	7486	80.1% (0.005)
Missing data	69 (0.7%)	
Sleep disturbance		
Rarely or never	2131	22.3% (0.006)
Sometimes	4404	45.9% (0.007)
Most of the time	3253	31.8% (0.005)
Missing data	40 (0.4%)	
Physical activity		
Moderate or vigorous > once a week	5668	62.1% (0.008)
Moderate or vigorous ≤1 week	4151	37.9% (0.008
Missing data	9 (0.09%)	
Physical Activity Index Score	9763	9.0 (0.12)
Missing data	65 (0.7%)	

*Unweighted frequencies represent sample counts and weighted proportion or mean (linearised SE) represents corresponding estimated population values (through sampling weights, 9828 represent 50 923 453 community-dwelling American adults aged 50 years and above).

†Major disease defined as having a history of cancer (excluding skin), lung disease, heart condition or stroke.

BMI, body mass index.

### Demographic and health-related characteristics and incident pain

The data on reported pain status were provided by 8513 (86.6%) respondents in 2016. After applying survey weights, an estimated 18.6% (SE=0.005) of the sampled population had self-reported incident troublesome pain in 2016. This was associated with female gender, older age, higher BMI, less education, being separated, divorced or widowed, poorer self-rated health, having a history of depression, major disease, Alzheimer’s disease or dementia, arthritis, diabetes, a higher frequency of sleep disturbance and lower levels of physical activity (table 2). Having missing data for pain status in 2016 was associated with older age, higher BMI, Hispanic ethnicity, less formal education, being widowed, poorer self-rated health, a history of major disease, Alzheimer’s disease or dementia and lower physical activity levels in 2014.

**Table 2:** Physical Activity Index Scores in 2014 and incident pain in 2016 by demographic and health-related factors

	2014 Physical Activity Index		2016 Incident pain	
	Weighted mean or B (SE)	P value	Weighted proportion or OR (SE)	P value
Gender				
Female	8.3 (0.1)	<0.001	20.1% (0.008)	0.01
Male	9.6 (0.2)		16.9% (0.008)	
Age	B=−0.2 (0.009)	<0.001	OR=1.01 (0.004)	0.03
BMI				
Underweight/normal weight	9.9 (0.2)	<0.001	15.4 (0.008)	<0.001
Overweight	9.5 (0.2)		16.8 (0.008)	
Obese	8.4 (0.2)		22.5 (0.01)	
Obese, BMI ≥35	6.4 (0.2)		26.7 (0.02)	
Race/ethnicity				
White	9.2 (0.1)	<0.001	17.9 (0.006)	0.06
Black	7.7 (0.2)		19.4 (0.01)	
Hispanic	8.4 (0.4)		23.6 (0.02)	
Other	8.7 (0.5)		19.4 (0.03)	
Years of school	B=0.5 (0.04)	<0.001	OR=0.9 (0.008)	<0.001
Marital status				
Married	9.6 (0.2)	<0.001	17.5% (0.007)	0.002
Separated or divorced	9.0 (0.2)		21.2% (SE=0.01)	
Widowed	6.7 (0.2)		21.6% (0.01)	
Never married	7.9 (0.3)		16.5% (0.03)	
Self-rated health				
Excellent	12.3 (0.3)	<0.001	9.0% (0.01)	<0.001
Very good	9.9 (0.2)		14.9% (0.006)	
Good	7.8 (0.1)		22.9% (0.01)	
Fair	6.0 (0.3)		30.2% (0.02)	
Poor	2.6 (0.2)		34.7% (0.03)	
History of depression				
Yes	7.9 (0.3)	<0.001	29.3% (0.01)	<0.001
No	9.2 (0.1)		16.3% (0.006)	
History of major disease*				
Yes	7.5 (0.1)	<0.001	22.8% (0.009)	<0.001
No	9.8 (0.2)		16.4% (0.007)	
Alzheimer’s disease or dementia				
Yes	4.1 (0.5)	<0.001	30.7% (0.03)	<0.001
No	9.1 (0.1)		18.4% (0.005)	
Arthritis				
Yes	8.2 (0.1)	<0.001	26.8% (0.007)	<0.001
No	9.5 (0.2)		12.3% (0.006)	
Diabetes				
Yes	7.1 (0.2)	<0.001	23.1% (0.01)	<0.001
No	9.4 (0.1)		17.5% (0.005)	
Sleep disturbance				
Rarely or never	9.9 (0.2)	<0.001	11.8% (0.01)	<0.001
Sometimes	9.0 (0.2)		17.0% (0.007)	
Most of the time	8.3 (0.2)		25.7% (0.01)	
2014 Physical Activity Index				
Moderate or vigorous >1 week			17.2% (0.006)	<0.001
Moderate or vigorous ≤1 week			21.0% (0.008)	
Physical Activity Index Score			OR=0.97 (0.004)	<0.001

*Major disease defined as having a history of cancer (excluding skin), lung disease, heart condition or stroke.

B, beta; BMI, body mass index.

## Physical activity

37.9% (SE=0.008) of the baseline population participated in moderate or vigorous physical activity once a week or less. Overall, the mean baseline Physical Activity Index Score was 9.0 (SE=0.12). For those participating in moderate or vigorous physical activity once a week or less, the mean Physical Activity Index Score was 2.5 (SE=0.07); for those who reported participating in moderate or vigorous activity more than once a week, the mean Physical Activity Index Score was 12.9 (SE=0.10).

## Sleep

22.3% (SE=0.006) of the baseline pain-free sample reported rarely or never having sleep disturbance, 45.9% (SE=0.007) reported sometimes experiencing sleep disturbance and 31.8% (SE=0.005) reported experiencing sleep disturbance most of the time.

### The association between physical activity and incident troublesome pain and the moderating effect of sleep disturbance

**Figure 1:**
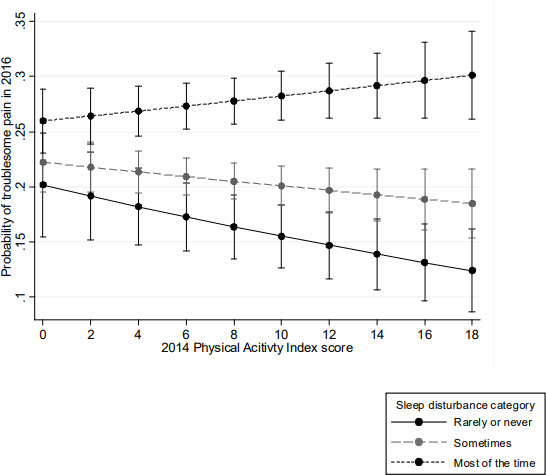
The moderating effect of sleep disturbance on the association between physical activity and later incident pain.

In models adjusted for pre-specified covariates and stratified by sleep disturbance categories, each one point higher on the Physical Activity Index Score (range=0–18) was associated with a reduced likelihood of troublesome pain onset for those who reported ‘never/rarely’ having sleep disturbance (OR=0.97, 95% CI 0.94 to 0.99). The protective effect attenuated with increasing frequency of sleep disturbance (sleep disturbance sometimes: OR=0.99, 95% CI 0.97 to 1.01; sleep disturbance most of the time: OR=1.01, 95% CI 0.99 to 1.03). In an adjusted model that included a Physical Activity Index Score–frequency of sleep disturbance interaction term, a significant moderating effect of sleep disturbance on the Physical Activity Index Score–incident pain association was observed (Wald test for the interaction: p=0.02) (table 3, figure 1).

**Table 3:** Logistic regression analysis investigating the moderating effect of sleep disturbance category on the association between 2014 Physical Activity Index Score and likelihood of troublesome pain in 2016*

	OR (95% CI)	Linearised SE	T	P
Physical Activity Index Score	0.97 (0.94 to 0.99)	0.01	−2.46	0.02
Sleep disturbance category				
Rarely or never	Reference			
Sometimes	1.14 (0.78 to 1.67)	0.22	0.68	0.50
Most of the time	1.42 (0.99 to 2.05)	0.26	1.92	0.06
Physical Activity Index Score–sleep disturbance category interaction term*				
Rarely or never	Reference			
Sometimes	1.02 (0.99 to 1.06)	0.02	1.18	0.24†
Most of the time	1.05 (1.01 to 1.08)	0.02	2.79	0.01†
Age	0.99 (0.99 to 1.00)	0.004	−1.57	0.12
Gender				
Male	Reference			
Female	1.02 (0.85 to 1.23)	0.10	0.20	0.84
BMI				
Underweight/normal weight	Reference			
Overweight	1.05 (0.88 to 1.26)	0.09	0.57	0.57
Obese	1.36 (1.09 to 1.70)	0.15	2.74	0.01
Obese, BMI ≥35	1.56 (1.22 to 1.99)	0.19	3.63	0.001
Race/ethnicity				
White	Reference			
Black	1.02 (0.85 to 1.22)	0.09	0.22	0.83
Hispanic	1.27 (0.92 to 1.77)	0.21	1.49	0.14
Other	1.28 (0.86 to 1.89)	0.25	1.25	0.22
Years of school	0.95 (0.93 to 0.97)	0.01	−5.13	<0.001
History of depression	1.69 (1.38 to 2.07)	0.17	5.17	<0.001
History of major disease‡	1.24 (1.02 to 1.51)	0.12	2.22	0.03
Arthritis	2.35 (2.00 to 2.78)	0.19	10.41	<0.001
Diabetes	1.05 (0.92 to 1.21)	0.07	0.73	0.47

*Adjusted analysis: N=8036.

†Wald test statistic for overall interaction: p=0.02.

‡Major disease defined as having a history of cancer (excluding skin), lung disease, heart condition or stroke.

BMI, body mass index.

## Discussion

This nationally representative prospective cohort of US adults shows that self-reported sleep disturbance moderates the association between physical activity and later pain. Although higher levels of physical activity were associated with reduced likelihood of incident troublesome pain among those who reported sleep disturbance never, rarely or sometimes, the benefit of higher levels of physical activity was not observed for those who reported disturbed sleep most of the time.

Previous cross-sectional research has demonstrated linear, negative associations between increasing levels of exercise frequency, duration and intensity and the presence of chronic pain.^17^ Our finding that increasing levels of physical activity are associated with a reduced likelihood of later pain reporting is consistent with this and, in addition to the prospective nature of our study, we have extended the evidence by identifying a moderating effect of sleep disturbance on this association. Given our results, it is possible that the beneficial influence of physical activity on pain reporting has previously been underestimated, and perhaps muted by a proportion having unmeasured or unaccounted for chronic sleep disturbance.

Mechanisms by which sleep disturbance may impact the association between level of physical activity and later pain onset are likely multidimensional,^41^ potentially acting as both moderators and mediators. Sleep disturbance, including delayed sleep onset, waking up during the night and waking up earlier than intended, can be associated with reduced total sleep time and, consequently, less time spent in non-rapid eye movement slow-wave sleep (SWS).^42^ Adequate SWS is thought to contribute to the recuperative function of sleep. A reduced duration of SWS and, therefore, protracted restorative sleep have been shown to reduce next-day performance^43^ and may diminish the beneficial effects of physical activity by limiting recovery. Suboptimal sleep also has a negative impact on immune function,^44 45^ and a body of evidence supports a case for sleep as essential to recovery after exercise in elite athletes.^46–48^ It is plausible that similar mechanisms may be at play for older adults who participate in higher levels of physical activity—within the context of disturbed sleep, the benefit of higher levels of physical activity may not only be weakened, but may render sleep-disturbed individuals more susceptible to poor health outcomes.

Focusing exclusively on a working population, Skarpsno and colleagues examined the role of both occupational and leisure time physical activity on later sleep disturbance.^49^ For those with pain, higher levels of objectively measured physical activity (both at work and during leisure time) were associated with a higher prevalence of sleep disturbance. Skarpsno and colleagues argue that higher levels of occupational physical activity may lead to an overload response^49^ (as described by Sluiter and colleagues,^50^) increasing the probability of sleep disturbance. We hypothesise that such an overload response may also be pertinent to our observation of the negative influence of sleep disturbance on the physical activity–pain relationship. In the absence of adequate restorative sleep, benefits of physical activity may be counterpointed by an aggregating overload response. However, with a population aged 50 years and above, the motivation driving different modes of physical activity may be salient; retirees who choose to participate in higher levels of physical activity may have very different experiences to those over 50 in employment, particularly those in physically demanding jobs.

We have investigated any sleep disturbance as a moderator of the physical activity–pain onset association. However, given that each individual sleep item could be argued as representing an independent sleep disturbance domain with different implications on pain reporting, in post-hoc analysis, we explored the potentially moderating effect of each specific sleep disturbance (trouble falling asleep, staying asleep, waking too early, and feeling unrefreshed on awakening). The moderating effect was most pronounced for trouble falling asleep, and all sleep disturbance domains exhibited a similar pattern of effect, with the exception that waking too early showed no sign of moderating the physical activity–pain onset association (online Supplementary file 1). Focused research into the moderating effect of specific sleep domains, including those not available in the HRS dataset (eg, sleep duration), are recommended in future research, where more granular data collection, including objective assessments, could help to comprehensively address questions about the impact of specific sleep domains on the physical activity–pain onset association.

10.1136/bmjopen-2019-036219.supp1 Supplementary data

### Strengths and limitations

Study strengths are the relatively large sample size, representativeness of the data to the American adult population ≥50 years of age and low attrition rates. The advantages of access to and use of this dataset, however, need to be balanced against unavoidable limitations. Sleep disturbance, physical activity and pain were all assessed by self-report and are, therefore, susceptible to recall bias. Given the broad nature of response categories for pain and sleep disturbances, measurement imprecision may have resulted in misclassification that could have biased the effect estimates. The HRS questionnaire uses different response categories to the Jenkins Sleep Scale and, in the absence of comparative data, the validity of these items remains open to question. Future validation study of HRS sleep item responses against the Jenkins Sleep Scale would benefit future sleep-related research that uses the HRS dataset. Our pain outcome variable specifically is broad brush (any troublesome pain, regardless of intensity or duration). However, the use of this outcome variable was based on previous research that suggests it is a good indicator of pain that interferes with daily activities.^35^ Furthermore, it has been shown that pain is often reported as mild by people with chronic pain^51 52^ and we would, therefore, have captured these cases using this single-item question.

Residual confounding cannot be excluded. We specifically chose not to take into account the consumption of medications for either sleep disturbance or pain in our analyses. From a pragmatic point of view, we were interested in problematic sleep and pain experiences—if they were being successfully managed, we considered them not to be a current problem. Also, although we adjusted for a history of depression, we did not have information on whether participants were taking antidepressant medications. Given potential effects on both sleep and pain reporting, collecting this information and including it in future analyses is recommended. An additional source of potential confounding given the age of the cohort is the built environment and living arrangements. In a post-hoc sensitivity analysis, we adjusted for nursing home status and living arrangements and the interpretation of our findings remained unchanged (online supplementary file 2). Finally, although prospective in design, this study only used two assessment time points. To provide greater insights into temporal processes, more intensive data collection and analysis would add value. Despite these limitations, our findings are consistent with existing evidence and, we believe, provide support for an argument to spend the time and finances required by further investigation.

10.1136/bmjopen-2019-036219.supp2 Supplementary data

Use of objective measures of sleep and physical activity that adhere to emerging guidelines^53^ is recommended. Investigation of the possible differential impact of type of physical activity and exercise (eg, leisure time or occupational physical activity; aerobic or strengthening exercise) may also prove illuminating. It may also be worthwhile to extend these investigations to clinical populations with pain (eg, musculoskeletal, cancer and multiple sclerosis) to examine whether the sleep–physical activity interaction is also pertinent to the persistence or worsening of pain intensity or interference.

## Conclusion

Sleep disturbance moderated the association between physical activity and incident troublesome pain in American adults aged 50 years and above. These findings warrant further examination. Replication would provide a strong case for integrating sleep health alongside physical activity recommendations for older adults to promote healthy ageing and prevention of pain onset.

## Supplementary Material

Reviewer comments

Author's manuscript
