# Supplementary material for: Sleep disturbance as a moderator of the association between physical activity and later pain onset among American adults aged 50 and over: evidence from the Health and Retirement Study
Source: BMJ Open. 2020 Jun 7;10(6):e036219. doi: 10.1136/bmjopen-2019-036219 (PMC7282328; doi:10.1136/bmjopen-2019-036219)

Supplement 1.

Table S1A. Logistic regression analysis investigating the moderating effect of having trouble falling asleep on the association between 2014 physical activity index score and likelihood of troublesome pain in 2016*

	**Odds Ratio (95% CI)**	**Linearized SE**	**T**	**P**
**Physical activity index score** (PAI)	0.98 (0.97–0.99)	0.007	-2.75	0.008
**Trouble falling asleep**				
Rarely or never	Reference			
Sometimes	1.15 (0.88–1.51)	0.15	1.07	0.29
Most of the time	1.26 (0.91–1.74)	0.20	1.41	0.16
**Physical activity index score * sleep disturbance category interaction term**				
Rarely or never	Reference			
Sometimes	1.02 (1.00–1.05)	0.01	1.88	0.07[a]
Most of the time	1.04 (1.00–1.07)	0.02	2.18	**0.03**[a]
**Age**	0.99 (0.98–1.00)	0.004	-1.54	0.13
**Gender**				
Male	Reference			
Female	0.97 (0.81–1.17)	0.09	-0.31	0.76
**BMI**				
Underweight/normal weight	Reference			
Overweight	1.05 (0.87–1.26)	0.09	0.50	0.62
Obese	1.36 (1.10–1.69)	0.15	2.84	0.006
Obese, BMI ≥35	1.55 (1.21–2.00)	0.19	3.53	0.001
**Race/ethnicity**				
White	Reference			
Black	0.98 (0.81–1.17)	0.09	-0.26	0.79
Hispanic	1.27 (0.92–1.75)	0.21	1.47	0.15
Other	1.23 (0.83–1.82)	0.24	1.07	0.29
**Years of school**	0.95 (0.93–0.97)	0.01	-4.73	<0.001
**History of depression**	1.69 (1.39–2.07)	0.17	5.31	<0.001
**History of major disease**[†]	1.24 (1.03–1.51)	0.12	2.28	0.03
**Arthritis**	2.39 (2.02–2.82)	0.20	10.51	<0.001
**Diabetes**	1.07 (0.93–1.22)	0.07	0.94	0.35

* Adjusted analysis N=8,037
CI: confidence interval; SE: standard error
[a] Wald test statistic for overall interaction: p=0.06
[†] Major disease defined as having a history of cancer (excluding skin), lung disease, heart condition, or stroke.

Table S1B. Logistic regression analysis investigating the moderating effect of having trouble waking up during the night on the association between 2014 physical activity index score and likelihood of troublesome pain in 2016*

	Odds Ratio (95% CI)	Linearized SE	T	P
**Physical activity index score** (PAI)	0.98 (0.96–0.99)	0.008	-2.61	0.01
**Trouble waking up during the night**				
Rarely or never	Reference			
Sometimes	1.08 (0.81–1.43)	0.15	0.51	0.61
Most of the time	1.36 (1.04–1.79)	0.19	2.29	0.03
**Physical activity index score * sleep disturbance category interaction term**				
Rarely or never	Reference			
Sometimes	1.01 (0.99–1.04)	0.01	1.24	0.22[a]
Most of the time	1.03 (1.00–1.06)	0.01	1.92	0.06[a]
**Age**	0.99 (0.98–1.00)	0.004	-1.79	0.08
**Gender**				
Male	Reference			
Female	1.02 (0.85–1.22)	0.09	0.19	0.85
**BMI**				
Underweight/normal weight	Reference			
Overweight	1.06 (0.88–1.27)	0.09	0.65	0.52
Obese	1.36 (1.09–1.70)	0.15	2.81	0.007
Obese, BMI ≥35	1.56 (1.22–1.99)	0.19	3.62	0.001
**Race/ethnicity**				
White	Reference			
Black	1.05 (0.88–1.26)	0.10	0.54	0.59
Hispanic	1.34 (0.97–1.87)	0.22	1.80	0.08
Other	1.32 (0.90–1.95)	0.26	1.43	0.16
**Years of school**	0.94 (0.92–0.96)	0.01	-5.58	<0.001
**History of depression**	1.74 (1.43–2.14)	0.18	5.49	<0.001
**History of major disease**[†]	1.25 (1.03–1.52)	0.12	2.34	0.02
**Arthritis**	2.36 (2.00–2.78)	0.19	10.40	<0.001
**Diabetes**	1.05 (0.91–1.20)	0.07	0.66	0.51

* Adjusted analysis N=8,018
CI: confidence interval; SE: standard error
[a] Wald test statistic for overall interaction: p=0.15
[†] Major disease defined as having a history of cancer (excluding skin), lung disease, heart condition, or stroke.

Table S1c. Logistic regression analysis investigating the moderating effect of having trouble with waking up too early on the association between 2014 physical activity index score and likelihood of troublesome pain in 2016*

	Odds Ratio (95% CI)	Linearized SE	T	P
**Physical activity index score** (PAI)	0.99 (0.98–1.00)	0.007	-1.30	0.20
**Trouble with waking up too early**				
Rarely or never	Reference			
Sometimes	1.26 (1.00–1.59)	0.15	1.98	0.05
Most of the time	1.79 (1.21–2.64)	0.35	3.00	0.004
**Physical activity index score * sleep disturbance category interaction term**				
Rarely or never	Reference			
Sometimes	1.00 (0.98–1.02)	0.01	0.26	0.79[a]
Most of the time	0.99 (0.96–1.03)	0.02	-0.46	0.65[a]
**Age**	0.99 (0.99–1.00)	0.004	-1.48	0.14
**Gender**				
Male	Reference			
Female	1.02 (0.85–1.23)	0.09	0.25	0.80
**BMI**				
Underweight/normal weight	Reference			
Overweight	1.05 (0.88–1.26)	0.10	0.57	0.57
Obese	1.37 (1.10–1.69)	0.15	2.93	0.005
Obese, BMI ≥35	1.55 (1.21–1.98)	0.19	3.55	0.001
**Race/ethnicity**				
White	Reference			
Black	1.00 (0.84–1.20)	0.09	0.04	0.97
Hispanic	1.27 (0.92–1.76)	0.21	1.47	0.15
Other	1.25 (0.85–1.84)	0.24	1.15	0.26
**Years of school**	0.95 (0.93–0.97)	0.01	-4.84	<0.001
**History of depression**	1.77 (1.45–2.16)	0.18	5.67	<0.001
**History of major disease**[†]	1.24 (1.03–1.50)	0.12	2.27	0.03
**Arthritis**	2.38 (2.02–2.81)	0.20	10.58	<0.001
**Diabetes**	1.07 (0.93–1.22)	0.07	0.98	0.33

* Adjusted analysis N=8,034
CI: confidence interval; SE: standard error
[a] Wald test statistic for overall interaction: p=0.83
[†] Major disease defined as having a history of cancer (excluding skin), lung disease, heart condition, or stroke.

Table S1D. Logistic regression analysis investigating the moderating effect of feeling unrested on waking on the association between 2014 physical activity index score and likelihood of troublesome pain in 2016*

	**Odds Ratio (95% CI)**	**Linearized SE**	**T**	**P**
**Physical activity index score** (PAI)	0.99 (0.98–1.00)	0.007	-1.70	0.10
**Feeling unrested on waking**				
Rarely or never	Reference			
Sometimes	1.36 (1.08–1.71)	0.16	2.67	0.01
Most of the time	1.46 (1.10–1.93)	0.21	2.69	0.009
**Physical activity index score * sleep disturbance category interaction term**				
Rarely or never	Reference			
Sometimes	1.00 (0.98–1.03)	0.01	0.12	0.90[a]
Most of the time	1.04 (1.00–1.07)	0.02	1.90	0.06[a]
**Age**	1.00 (0.99–1.00)	0.004	-0.80	0.43
**Gender**				
Male	Reference			
Female	1.03 (0.86–1.23)	0.09	0.37	0.72
**BMI**				
Underweight/normal weight	Reference			
Overweight	1.05 (0.88–1.26)	0.09	0.58	0.57
Obese	1.36 (1.10–1.69)	0.15	2.88	0.006
Obese, BMI ≥35	1.55 (1.20–1.99)	0.19	3.49	0.001
**Race/ethnicity**				
White	Reference			
Black	0.98 (0.82–1.18)	0.09	-0.18	0.86
Hispanic	1.22 (0.88–1.69)	0.20	1.21	0.23
Other	1.27 (0.87–1.87)	0.25	1.25	0.22
**Years of school**	0.95 (0.93–0.97)	0.01	-4.96	<0.001
**History of depression**	1.69 (1.37–2.09)	0.18	4.98	<0.001
**History of major disease**[†]	1.24 (1.02–1.51)	0.12	2.21	0.03
**Arthritis**	2.37 (2.02–2.79)	0.19	10.65	<0.001
**Diabetes**	1.06 (0.93–1.21)	0.07	0.86	0.39

* Adjusted analysis N=8,041
CI: confidence interval; SE: standard error
[a] Wald test statistic for overall interaction: p=0.17
[†] Major disease defined as having a history of cancer (excluding skin), lung disease, heart condition, or stroke.

Figure S1. The moderating effect of sleep disturbance domains on the association between physical activity and later incident pain

A. Trouble falling asleep

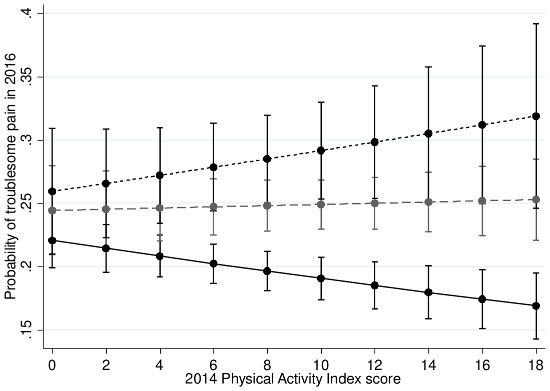

B. Trouble waking up during the night

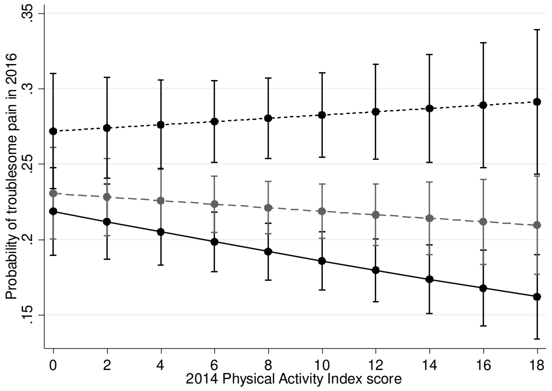

C. Trouble with waking up too early

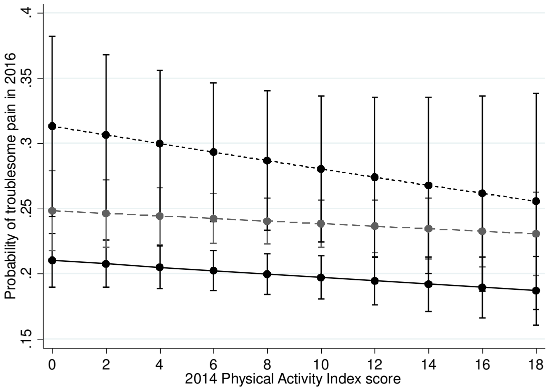

D. Feeling unrested on waking

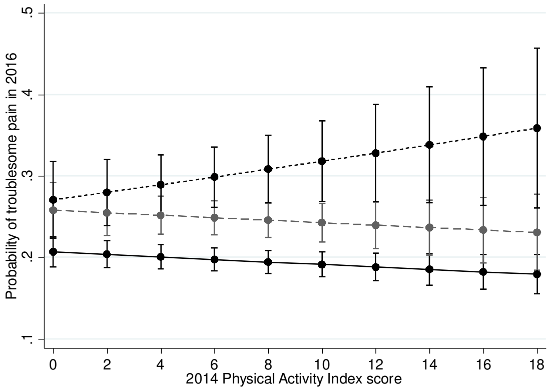

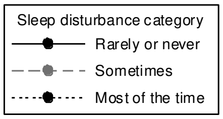

Supplement: Supplementary data [file bmjopen-2019-036219supp001.pdf]
