# Supplementary material for: Sleep disturbance as a moderator of the association between physical activity and later pain onset among American adults aged 50 and over: evidence from the Health and Retirement Study
Source: BMJ Open. 2020 Jun 7;10(6):e036219. doi: 10.1136/bmjopen-2019-036219 (PMC7282328; doi:10.1136/bmjopen-2019-036219)
Supplement: Supplementary data [file bmjopen-2019-036219supp002.pdf]

Supplement 2. Logistic regression analysis investigating the moderating effect of sleep disturbance category on the association between 2014 physical activity index score and likelihood of troublesome pain in 2016*, with additional adjustment for living arrangement status**

	OR (95% CI)	SE	T	P
**Physical activity index score** (PAI)	0.97 (0.94–0.99)	0.01	-2.44	0.02
**Sleep disturbance category**				
Rarely or never	Reference			
Sometimes	1.14 (0.78–1.67)	0.22	0.69	0.50
Most of the time	1.43 (0.99–2.06)	0.26	1.95	0.06
**Physical activity index score * sleep disturbance category interaction term**				
Rarely or never	Reference			
Sometimes	1.02 (0.99–1.06)	0.02	1.17	0.25[a]
Most of the time	1.05 (1.01–1.08)	0.02	2.77	0.01[a]
**Age**	0.99 (0.99–1.00)	0.004	-1.47	0.15
**Gender**				
Male	Reference			
Female	1.02 (0.84–1.25)	0.10	0.23	0.82
**BMI**				
Underweight/normal weight	Reference			
Overweight	1.05 (0.88–1.26)	0.09	0.60	0.55
Obese	1.36 (1.09–1.70)	0.15	2.74	0.01
Obese, BMI ≥35	1.57 (1.23–2.01)	0.19	3.66	0.001
**Race/ethnicity**				
White	Reference			
Black	1.02 (0.84–1.23)	0.10	0.18	0.86
Hispanic	1.26 (0.91–1.75)	0.21	1.40	0.17
Other	1.27 (0.86–1.89)	0.25	1.23	0.22
**Years of school**	0.95 (0.93–0.97)	0.01	-5.15	<0.001
**History of depression**	1.69 (1.38–2.06)	0.17	5.19	<0.001
**History of major disease**†	1.24 (1.02–1.51)	0.12	2.19	0.03
**Arthritis**	2.35 (1.99–2.77)	0.19	10.38	<0.001
**Diabetes**	1.05 (0.91–1.20)	0.07	0.71	0.48
**Living arrangement status****				
Married or partnered, living with partner	Reference			
Married or partnered, living with partner	1.22 (0.80–1.86)	0.26	0.94	0.35
Not married or partnered, living with unrelated adult	1.13 (0.50–2.58)	0.47	0.31	0.76
Not married or partnered, living with relative	0.99 (0.76–1.29)	0.13	-0.07	0.95
Not married or partnered, living alone	0.97 (0.79–1.19)	0.10	-0.32	0.75
Unknown	1.06 (0.24–4.75)	0.79	0.08	0.94

* Adjusted analysis N=8,036
OR: Odds Ratio; CI: confidence interval; SE: linearized standard error
[a] Wald test statistic for overall interaction: p=0.02
† Major disease defined as having a history of cancer (excluding skin), lung disease, heart condition, or stroke.
**Nursing home status omitted due to collinearity with living arrangement status. Of the total baseline sample, N= 16,361 were interviewed in the community and N=23 were interviewed in a nursing home in 2014. Of those interviewed in a nursing home, N=7 were also categorized within living arrangement status as 'married or partnered, living with partner'; N=16 were also categorized as 'not married or partnered, living alone'.
