# Supplementary material for: Sleep disturbance as a moderator of the association between physical activity and later pain onset among American adults aged 50 and over: evidence from the Health and Retirement Study
Source: BMJ Open. 2020 Jun 7;10(6):e036219. doi: 10.1136/bmjopen-2019-036219 (PMC7282328; doi:10.1136/bmjopen-2019-036219)
Supplement: Author's manuscript [file bmjopen-2019-036219.draft_revisions.pdf]

BMJ Open

BMJ Open is committed to open peer review. As part of this commitment we make the peer review history of every article we publish publicly available.

When an article is published we post the peer reviewers' comments and the authors' responses online. We also post the versions of the paper that were used during peer review. These are the versions that the peer review comments apply to.

The versions of the paper that follow are the versions that were submitted during the peer review process. They are not the versions of record or the final published versions. They should not be cited or distributed as the published version of this manuscript.

BMJ Open is an open access journal and the full, final, typeset and author-corrected version of record of the manuscript is available on our site with no access controls, subscription charges or pay-per-view fees (http://bmjopen.bmj.com).

If you have any questions on BMJ Open's open peer review process please email info.bmjopen@bmj.com

Sleep disturbance moderates the association between physical activity and pain onset in older American adults

Journal:	*BMJ Open*
Manuscript ID	bmjopen-2019-036219
Article Type:	Original research
Date Submitted by the Author:	05-Dec-2019

Complete List of Authors:	Whibley, Daniel; University of Aberdeen, ; University of Michigan,<br>Guyer, Heidi; University of Michigan,<br>Swanson, Leslie; University of Michigan,<br>Braley, Tiffany; University of Michigan,<br>Kratz, A; University of Michigan,<br>Dunietz, Galit; University of Michigan,

Keywords:	EPIDEMIOLOGY, PUBLIC HEALTH, PAIN MANAGEMENT

SCHOLARONE™
Manuscripts

I, the Submitting Author has the right to grant and does grant on behalf of all authors of the Work (as defined in the below author licence), an exclusive licence and/or a non-exclusive licence for contributions from authors who are: i) UK Crown employees; ii) where BMJ has agreed a CC-BY licence shall apply, and/or iii) in accordance with the terms applicable for US Federal Government officers or employees acting as part of their official duties; on a worldwide, perpetual, irrevocable, royalty-free basis to BMJ Publishing Group Ltd ("BMJ") its licensees and where the relevant Journal is co-owned by BMJ to the co-owners of the Journal, to publish the Work in this journal and any other BMJ products and to exploit all rights, as set out in our licence.

The Submitting Author accepts and understands that any supply made under these terms is made by BMJ to the Submitting Author unless you are acting as an employee on behalf of your employer or a postgraduate student of an affiliated institution which is paying any applicable article publishing charge ("APC") for Open Access articles. Where the Submitting Author wishes to make the Work available on an Open Access basis (and intends to pay the relevant APC), the terms of reuse of such Open Access shall be governed by a Creative Commons licence – details of these licences and which Creative Commons licence will apply to this Work are set out in our licence referred to above.

Other than as permitted in any relevant BMJ Author's Self Archiving Policies, I confirm this Work has not been accepted for publication elsewhere, is not being considered for publication elsewhere and does not duplicate material already published. I confirm all authors consent to publication of this Work and authorise the granting of this licence.

For peer review only

Title:

Sleep disturbance moderates the association between physical activity and pain onset in older American adults

Authors: Daniel Whibley[1,2], Heidi M. Guyer[3], Leslie M. Swanson[4], Tiffany J. Braley[5], Anna L. Kratz[2], Galit Levi Dunietz[6]

Affiliations:

1. Epidemiology Group, School of Medicine, Medical Sciences and Nutrition, University of Aberdeen, Scotland, UK

2. Department of Physical Medicine and Rehabilitation, University of Michigan, Ann Arbor, MI

3. Survey Research Center. Institute for Social Research. University of Michigan, Ann Arbor, MI. RTI International, North Carolina.

4. Department of Psychiatry, University of Michigan, Ann Arbor, MI

5. Department of Neurology, Division of Multiple Sclerosis and Neuroimmunology, University of Michigan, Ann Arbor, MI

6. Department of Neurology, Division of Sleep Medicine, University of Michigan, Ann Arbor, MI

Corresponding author: Daniel Whibley, PhD, Department of Physical Medicine & Rehabilitation, University of Michigan, North Campus Research Complex, 2800 Plymouth Rd, Building NCRC B16, Ann Arbor, MI, USA; daniel.whibley@abdn.ac.uk

Keywords: Physical activity; sleep disturbance; pain; older adults

For peer review only - http://bmjopen.bmj.com/site/about/guidelines.xhtml

Abstract

Objective: To examine whether sleep disturbance modifies the association between physical activity and incident pain

Design: Prospective population-based study.

Setting: Health and Retirement Study (HRS).

Participants: American adults aged ≥50 years who reported no troublesome pain in 2014 were re-assessed for pain in 2016. Of 9828 eligible baseline respondents, 8036 (82%) had complete follow-up data for adjusted analyses (weighted analysis population N=42,407,222).

Exposures: Physical activity, assessed via interview with questions about time spent in moderate and vigorous physical activity. Sleep disturbance, assessed using a modified form of the Jenkins Sleep Scale, was examined as a potential moderator.

Main Outcome measure: Troublesome pain.

Results: In weighted analyses, 37.9% of the 2014 baseline pain-free sample participated in moderate or vigorous physical activity ≤ once a week, with an overall mean physical activity index score of 9.0 (SE 0.12). 18.6% went on to report troublesome pain in 2016. Each one-point higher on the physical activity index score was associated with a reduced odds of incident pain for those who endorsed sleep disturbance never/rarely (OR 0.97, 95%CI 0.94–0.99), but not for those who endorsed sleep disturbance sometimes (OR 0.99, 95%CI 0.97–1.01) or most of the time (OR 1.01, 95%CI 0.99–1.03). Analysis of possible interaction demonstrated that frequency of sleep disturbance moderated the physical activity-incident pain association (Wald test p=0.02).

Conclusions: The beneficial association of physical activity on reduced likelihood of later pain was only observed in persons who endorsed low levels of sleep disturbance.

For peer review only - http://bmjopen.bmj.com/site/about/guidelines.xhtml

Article summary: **Strengths and limitations of this study**

- This study uses data representative of the US population aged 50 years and over to provide evidence for the moderating effect of sleep disturbance on the association between level of physical activity and later pain onset.
- Strengths of the study include its complex survey design which supports population-level inference, and the low level of missing data.
- Limitations of the study include the self-reported nature of the variables under study, and the lack of available information on the presence of specific sleep disorders.

For peer review only - http://bmjopen.bmj.com/site/about/guidelines.xhtml

For peer review only

Introduction

Pain is a preeminent public health issue[1], affecting approximately 20% of the global adult population[2]. Its reach is pervasive, impacting on daily functioning[3], mental health[4], and quality of life[5]. The societal burden of pain is also vast, with annual economic costs, including those associated with healthcare and social services, exceeding $560 billion in the US alone[6]. Half of the older adult population in the US are estimated to experience troublesome pain[7]. With an increasingly older population[8] and observed associations between pain onset and a reduction in healthy ageing[9], discovery of strategies to prevent or reduce the proliferation of pain in older adults to support healthy ageing is imperative. The identification of modifiable and protective lifestyle factors that contribute to pain is key to guide population-level efforts to achieve this aim.

One such modifiable factor, and an important determinant of healthy ageing, is physical activity[10]. Physical activity is associated with lower levels of functional disability[11] and morbidity[12], better mental health[13-15], and a reduced likelihood of pain reporting[16,17]. Accordingly, physical activity is a principal health behavior included in the World Health Organization's (WHO) 2015 World Report on Ageing and Health[18].

Like low levels of physical activity, poor sleep quality and short sleep duration are associated with more functional disability[19], a wide range of diseases[20-23], poor mental health[24] and pain reporting[25-28]. Poor sleep quality and lower sleep efficiency are also associated with lower levels of physical activity and a decreased likelihood of uptake or maintenance of routine exercise[29]. Conversely, higher levels of physical activity are associated with improvements in subjectively and objectively measured sleep[30,31]. Despite this, advice about sleep behavior is rarely included in

For peer review only

physical activity guidance for older adults. A possible reason for this may be a tacit societal agreement that parameters of sleep (e.g. duration, timing and continuity) inevitably decline with age. However, although changes are observed as humans age, most changes occur during the transition from young to middle adulthood[32]. Indeed, the National Sleep Foundation recommends that older adults sleep 7-8 hours per night (the recommendation for adults aged 18-64 is 7-9 hours)[33].

This study is predicated on the hypothesis that healthy sleep contributes to healthy ageing, and that sleep is an essential component, not only in its own right, but also as a condition within which the benefits of physical activity can be optimally reaped. To investigate the joint impact of sleep and physical activity on health, we examined whether sleep disturbance modified the association between level of physical activity and likelihood of incident troublesome pain two years later in a nationally representative sample of older adults in the US.

Methods

Study design

We used data from the Health and Retirement Study (HRS), a prospective survey of US adults aged 50 years and older conducted by the University of Michigan. Household face-to-face or telephone interviews are conducted with participants on a biannual basis[34]. Data collection is conducted by trained interviewers using laptop computers. The complex survey design of HRS incorporates multistage area probability sampling, stratification, clustering and oversampling of adults of African American and Hispanic race/ethnicity. This allows HRS to provide a nationally representative sample that, with appropriate design-based analyses, can be used to make

For peer review only - http://bmjopen.bmj.com/site/about/guidelines.xhtml

population-level inferences. A dataset comprising two HRS waves (2014 and 2016) was compiled and used to investigate relationships between physical activity, sleep disturbance, and incident pain (those with no pain in 2014 followed forward for pain status in 2016). The HRS is sponsored by the National Institute on Aging (grant number NIA U01AG009740). All data collection protocols are approved by the University of Michigan Institutional Review Board.

Measures

Pain

The outcome was incident troublesome pain, determined by a "yes" response to the question, "Are you often troubled with pain?" in 2016. This item has previously been shown to be comparable to pain that at least moderately interferes with daily life[35].

Physical activity

The World Health Organization (WHO) recommends that adults aged ≥65 years participate in 150 minutes of moderate physical activity or 75 minutes of vigorous physical activity each week, or an equivalent combination of both[36]. The physical health section of HRS surveys include questions about current levels of moderate and vigorous physical activity (Moderate: "how often do you take part in sports or activities that are moderately energetic such as, gardening, cleaning the car, walking at a moderate pace, dancing, floor or stretching exercises"; Vigorous: "How often do you take part in sports or activities that are vigorous, such as running or jogging, swimming, cycling, aerobics or gym workout, tennis, or digging with a spade or shovel"). Response options available for both questions are: hardly ever or never, one to three times a month, once a week, or more than once a week. Responses were used to create a physical activity index score, calculated following a previously used method[37,38]. Moderate activity responses were coded as: 0= hardly

For peer review only - http://bmjopen.bmj.com/site/about/guidelines.xhtml

ever, 1=one to three times a month, 3=once a week, 6=more than once a week. Vigorous activity responses were coded as: 0= hardly ever, 2=one to three times a month, 6=once a week, 12=more than once a week. A total physical activity index score was calculated by summing scored responses to these two questions (possible range 0–18). To provide context for physical activity index scores, a binary variable was also created, guided by WHO physical activity recommendations. Respondents were categorized as meeting the recommended level of physical activity if they participated in moderate activity more than once a week or every day, or vigorous activity more than once a week or every day. They were categorized as not meeting the recommendations if they did not participate in this amount of physical activity. According to this approach, physical activity index scores lower than 6 were indicative of physical inactivity; scores greater than 9 suggested meeting or exceeding WHO physical activity recommendations.

Sleep disturbance

Four questions about sleep disturbance type and frequency, adapted from the Jenkins Sleep Scale[39], were included in the physical health section of HRS: How often do you have trouble falling asleep?; How often do you have trouble with waking up during the night?; How often do you have trouble with waking up too early and not being able to fall asleep again?; How often do you feel really rested when you wake up in the morning? Response options were: rarely or never; sometimes; or most of the time (reverse coded for the question about feeling rested on awakening to ensure consistency with other questions). Respondents were categorized as never or rarely experiencing sleep disturbance if they answered rarely or never to all questions; sometimes, if they answered sometimes but not 'most of the time' to any of these questions, and most of the time if they answered 'most of the time' to any of these questions.

For peer review only - http://bmjopen.bmj.com/site/about/guidelines.xhtml

Demographic and Health Characteristics

Age, gender, race/ethnicity, and number of years of school education were included as demographic covariates. Five health-related covariates were also adjusted for: Body mass index (BMI), self-reported history of depression ("Has a doctor ever told you that you have had problems with depression?"), major disease (self-reported history of cancer (any kind except skin), lung disease, heart condition, or stroke), diabetes and arthritis.

Statistical analysis

Analyses were conducted using Stata (version 15.1, StataCorp LLC, College Station, TX). HRS sample weights were used to correct for unequal probability of selection that may have arisen as a result of the multistage sampling design. Demographic and health-related characteristics were summarized (mean, standard error). To investigate whether sleep disturbance reported in 2014 moderated the relationship between level of physical activity at this time and incident pain in 2016, logistic regression was performed with a physical activity index-sleep disturbance status interaction term. As the research question focused on those with no troublesome pain at baseline, subpopulation estimation was used to compute point and variance estimates[40]. This ensured that those reporting no pain at baseline contributed data to the calculation of effect estimates while those with and without pain at baseline contributed data to calculation of standard errors. In the presence of significant interaction ($p<0.05$), predicted probabilities of reporting pain at follow-up were calculated and plotted according to physical activity index score and sleep disturbance status. Models were adjusted for age, gender, race/ethnicity, number of years of school education, BMI, self-reported history of depression, major disease (self-reported history of cancer (any kind except skin), lung disease, heart condition, or stroke), diabetes and arthritis.

For peer review only - http://bmjopen.bmj.com/site/about/guidelines.xhtml

Patient and public involvement

Patients and/or members of the public were not involved in this study and there are no plans to disseminate the findings to HRS participants.

Results

Descriptive analysis

Of 16,384 HRS respondents, 16,330 (99.7%) answered the question about troublesome pain in 2014. Of these, 9,828 (60.2%) reported not being troubled by pain. Baseline demographic and health-related characteristics of this pain-free sample are presented in Table 1. After applying sample weights, 51.5% were female, and the mean age was 66.9.

Demographic and health-related characteristics and incident pain

8,513 (86.6%) provided data on pain status in 2016. An estimated 18.6% (SE 0.005) of the population reported incident troublesome pain. This was associated with female gender, older age, higher BMI, less education, being separated, divorced or widowed, poorer self-rated health, having a history of depression, major disease, Alzheimer's Disease or dementia, arthritis, diabetes, a higher frequency of sleep disturbance, and lower levels of physical activity (Table 2). Having missing data for pain status in 2016 was associated with older age, higher BMI, Hispanic ethnicity, less formal education, being widowed, poorer self-rated health, a history of major disease, Alzheimer's disease or dementia, and lower physical activity levels in 2014.

Physical activity

For peer review only - http://bmjopen.bmj.com/site/about/guidelines.xhtml

37.9% (SE 0.008) of the baseline population participated in moderate or vigorous physical activity once a week or less. Overall, the mean baseline physical activity index score was 9.0 (SE 0.12). For those participating in moderate or vigorous physical activity once a week or less, the mean physical activity index score was 2.5 (SE 0.07); for those who reported participating in moderate or vigorous activity more than once a week or every day, the mean physical activity index score was 12.9 (SE 0.10).

Sleep

22.3% (SE 0.006) of the baseline pain-free sample reported rarely or never having sleep disturbance, 45.9% (SE 0.007) reported sometimes experiencing sleep disturbance, and 31.8% (SE 0.005) reported experiencing sleep disturbance most of the time.

The association between physical activity and incident troublesome pain and the moderating effect of sleep disturbance

In models adjusted for pre-specified covariates and stratified by sleep disturbance categories, each one point higher on the physical activity index score (range 0–18) was associated with a reduced likelihood of troublesome pain onset for those who reported "never/rarely" having sleep disturbance (OR 0.97, 95%CI 0.94–0.99). The effect attenuated with increasing frequency of sleep disturbance (sleep disturbance sometimes: OR 0.99, 95%CI 0.97–1.01; sleep disturbance most of the time: OR 1.01, 95%CI 0.99–1.03). In an adjusted model that included a physical activity index score-frequency of sleep disturbance interaction term, a significant moderating effect of sleep disturbance on the physical activity index score-incident pain association was observed (Wald test for the interaction: p=0.02) (Table 3, Figure 1).

For peer review only - http://bmjopen.bmj.com/site/about/guidelines.xhtml

For peer review only

Table 1: Demographic and health characteristics of Health and Retirement Study (HRS) respondents not troubled by pain in 2014

	Unweighted frequency (N)[a]	Weighted proportion or mean (SE)[a]
Gender		
Female	5346	51.5% (0.005)
Male	4482	48.5% (0.005)
Age (mean, SE)	9828	66.9 (0.3)
BMI		
Underweight/normal weight	2816	31.2% (0.006)
Overweight	3426	38.9% (0.007)
Obese	1820	20.1% (0.006)
Obese, BMI ≥35	920	9.9% (0.004)
Missing data	846 (8.6%)	
Race/ethnicity		
White	6371	78.7% (0.01)
Black	1837	9.6% (0.006)
Hispanic	1301	8.4% (0.01)
Other	319	3.4% (0.003)
Years of school (mean, SE)	9792	13.5 (0.08)
Missing data	36 (0.4%)	
Marital status in 2014		
Married	5759	62.6% (0.007)
Separated or divorced	1644	16.2% (0.005)
Widowed	1943	14.4% (0.004)
Never married	481	6.8% (0.004)
Missing data	1 (0.01%)	
Self-rated health		
Excellent	1131	14.0% (0.005)
Very good	3611	40.6% (0.008)
Good	3255	30.7% (0.007)
Fair	1499	12.0% (0.006)
Poor	323	2.5% (0.002)
Don't know/refused	9 (0.09%)	
History of depression		
Yes	1554	16.8% (0.005)
No	8203	83.2% (0.005)
Missing data	71 (0.7%)	
History of major disease†		
Yes	3766	35.5% (0.008)
No	5960	64.5% (0.008)
Missing data	102 (1.0%)	
Alzheimer's Disease or dementia		
Yes	274	2.0% (0.001)
No	9510	98.0% (0.001)
Missing data	44 (0.5%)	

For peer review only - http://bmjopen.bmj.com/site/about/guidelines.xhtml

Arthritis		
Yes	4653	43.8% (0.008)
No	5175	56.2% (0.008)
Diabetes		
Yes	2273	19.9% (0.005)
No	7486	80.1% (0.005)
Missing data	69 (0.7%)	
Sleep disturbance		
Rarely or never	2131	22.3% (0.006)
Sometimes	4404	45.9% (0.007)
Most of the time	3253	31.8% (0.005)
Missing data	40 (0.4%)	
Physical activity		
Moderate or vigorous >1 week	5668	62.1% (0.008)
Moderate or vigorous ≤1 week	4151	37.9% (0.008
Missing data	9 (0.09%)	
Physical activity index score	9763	9.0 (0.12)
Missing data	65 (0.7%)	

[a] Unweighted frequencies represent sample counts; weighted proportion or mean (linearized SE) represent corresponding estimated population values (through sampling weights, 9,828 represent 50,923,453 community-dwelling American adults aged 50 years and above).

† Major disease defined as having a history of cancer (excluding skin), lung disease, heart condition, or stroke.

For peer review only

Table 2. Physical activity index scores in 2014 and incident pain in 2016 by demographic and health-related factors

	2014 Physical Activity Index		2016 Incident pain	
	Weighted mean or Beta, (SE)	p value	Weighted proportion or Odds Ratio, (SE)	p value
Gender				
Female	8.3 (0.1)	<0.001	20.1% (0.008)	
Male	9.6 (0.2)		16.9% (0.008)	0.01
Age	B = -0.2 (0.009)	<0.001	OR = 1.01 (0.004)	0.03
BMI				
Underweight/normal weight	9.9 (0.2)		15.4 (0.008)	
Overweight	9.5 (0.2)		16.8 (0.008)	
Obese	8.4 (0.2)		22.5 (0.01)	
Obese, BMI ≥35	6.4 (0.2)	<0.001	26.7 (0.02)	<0.001
Race/ethnicity				
White	9.2 (0.1)		17.9 (0.006)	
Black	7.7 (0.2)		19.4 (0.01)	
Hispanic	8.4 (0.4)		23.6 (0.02)	
Other	8.7 (0.5)	<0.001	19.4 (0.03)	0.06
Years of school	B = 0.5 (0.04)	<0.001	OR = 0.9 (0.008)	<0.001
Marital status				
Married	9.6 (0.2)		17.5% (0.007)	
Separated or divorced	9.0 (0.2)		21.2% (SE 0.01)	
Widowed	6.7 (0.2)		21.6% (0.01)	
Never married	7.9 (0.3)	<0.001	16.5% (0.03)	0.002
Self-rated health				
Excellent	12.3 (0.3)		9.0% (0.01)	
Very good	9.9 (0.2)		14.9% (0.006)	
Good	7.8 (0.1)		22.9% (0.01)	
Fair	6.0 (0.3)		30.2% (0.02)	
Poor	2.6 (0.2)	<0.001	34.7% (0.03)	<0.001
History of depression				
Yes	7.9 (0.3)		29.3% (0.01)	
No	9.2 (0.1)	<0.001	16.3% (0.006)	<0.001
History of major disease†				
Yes	7.5 (0.1)		22.8% (0.009)	
No	9.8 (0.2)	<0.001	16.4% (0.007)	<0.001
Alzheimer's Disease or dementia				
Yes	4.1 (0.5)		30.7% (0.03)	
No	9.1 (0.1)	<0.001	18.4% (0.005)	<0.001
Arthritis				
Yes	8.2 (0.1)		26.8% (0.007)	
No	9.5 (0.2)	<0.001	12.3% (0.006)	<0.001
Diabetes				
Yes	7.1 (0.2)		23.1% (0.01)	

For peer review only - http://bmjopen.bmj.com/site/about/guidelines.xhtml

No	9.4 (0.1)	<0.001	17.5% (0.005)	<0.001
Sleep disturbance				
Rarely or never	9.9 (0.2)		11.8% (0.01)	
Sometimes	9.0 (0.2)		17.0% (0.007)	
Most of the time	8.3 (0.2)	<0.001	25.7% (0.01)	<0.001
2014 Physical Activity Index				
Moderate or vigorous >1 week			17.2% (0.006)	
Moderate or vigorous ≤1 week			21.0% (0.008)	<0.001
Physical activity index score			OR = 0.97 (0.004)	<0.001

† Major disease defined as having a history of cancer (excluding skin), lung disease, heart condition, or stroke.

For peer review only

For peer review only - http://bmjopen.bmj.com/site/about/guidelines.xhtml

Table 3. Logistic regression analysis investigating the moderating effect of sleep disturbance category on the association between 2014 physical activity index score and likelihood of troublesome pain in 2016

	Odds Ratio (95% CI)	Linearized SE	T	P
**Physical activity index score** (PAI)	0.97 (0.94–0.99)	0.01	-2.46	0.02
**Sleep disturbance category**				
Rarely or never	Reference			
Sometimes	1.14 (0.78–1.67)	0.22	0.68	0.50
Most of the time	1.42 (0.99–2.05)	0.26	1.92	0.06
**Physical activity index score * sleep disturbance category interaction term**				
Rarely or never	Reference			
Sometimes	1.02 (0.99–1.06)	0.02	1.18	0.24[a]
Most of the time	1.05 (1.01–1.08)	0.02	2.79	0.01[a]
**Age**	0.99 (0.99–1.00)	0.004	-1.57	0.12
**Gender**				
Male	Reference			
Female	1.02 (0.85–1.23)	0.10	0.20	0.84
**BMI**				
Underweight/normal weight	Reference			
Overweight	1.05 (0.88–1.26)	0.09	0.57	0.57
Obese	1.36 (1.09–1.70)	0.15	2.74	0.01
Obese, BMI ≥35	1.56 (1.22–1.99)	0.19	3.63	0.001
**Race/ethnicity**				
White	Reference			
Black	1.02 (0.85–1.22)	0.09	0.22	0.83
Hispanic	1.27 (0.92–1.77)	0.21	1.49	0.14
Other	1.28 (0.86–1.89)	0.25	1.25	0.22
**Years of school**	0.95 (0.93–0.97)	0.01	-5.13	<0.001
**History of depression**	1.69 (1.38–2.07)	0.17	5.17	<0.001
**History of major disease**[†]	1.24 (1.02–1.51)	0.12	2.22	0.03
**Arthritis**	2.35 (2.00–2.78)	0.19	10.41	<0.001
**Diabetes**	1.05 (0.92–1.21)	0.07	0.73	0.47

CI: confidence interval; SE: standard error

[a] Wald test statistic for overall interaction: p=0.02

[†] Major disease defined as having a history of cancer (excluding skin), lung disease, heart condition, or stroke.

For peer review only - http://bmjopen.bmj.com/site/about/guidelines.xhtml

Discussion

This nationally representative prospective cohort of US adults shows that self-reported sleep disturbance moderates the association between physical activity and later pain. While higher levels of physical activity were associated with reduced likelihood of incident troublesome pain among those who reported sleep disturbance never, rarely, or sometimes, the benefit of higher levels of physical activity were not observed for those who reported disturbed sleep most of the time.

Previous cross-sectional research has demonstrated linear, negative associations between increasing levels of exercise frequency, duration and intensity and the presence of chronic pain[17]. Our finding that increasing levels of physical activity are associated with a reduced likelihood of later pain reporting is consistent with this and, in addition to the prospective nature of our study, we have extended the evidence by identifying a moderating effect of sleep disturbance on this association. Indeed, given our results, it is possible that the beneficial influence of physical activity on pain reporting has previously been underestimated, and perhaps muted by a proportion having unmeasured or unaccounted for chronic sleep disturbance.

Mechanisms by which sleep disturbance may moderate the association between level of physical activity and later pain onset are likely multidimensional[41]. Sleep disturbance, including delayed sleep onset, waking up during the night, and waking up earlier than intended, can be associated with reduced total sleep time and, consequently, less time spent in non-rapid eye movement slow-wave sleep (SWS)[42]. Adequate SWS is thought to contribute to the recuperative function of

For peer review only - http://bmjopen.bmj.com/site/about/guidelines.xhtml

sleep. A reduced duration of SWS and therefore protracted restorative sleep, has been shown to reduce next-day performance[43] and may diminish the beneficial effects of physical activity by limiting recovery. Suboptimal sleep also has a negative impact on immune function[44,45], and a body of evidence supports a case for sleep as essential to recovery after exercise in elite athletes[46-48]. It is plausible that similar mechanisms may be at play for older adults who participate in higher levels of physical activity – within the context of disturbed sleep the benefit of higher levels of physical activity may not only be weakened, but may render sleep-disturbed individuals more susceptible to poor health outcomes.

Focusing exclusively on a working population, Skarpsno and colleagues examined the role of both occupational and leisure time physical activity on later sleep disturbance[50]. For those with pain, higher levels of objectively measured physical activity (both at work and during leisure time) were associated with a higher prevalence of sleep disturbance. Skarpsno and colleagues argue that higher levels of occupational physical activity may lead to an overload response[51], increasing the probability of sleep disturbance. Such an overload response may also be pertinent to our observation of the negative influence of sleep disturbance on the physical activity-pain relationship. In the absence of adequate restorative sleep, benefits of physical activity may be counterpointed by an aggregating overload response. However, with a population aged 50 years and above, the motivation driving different modes of physical activity may be salient; retirees who choose to participate in higher levels of physical activity may have very different experiences to those over 50 in employment, particularly those in physically demanding jobs. Our analyses were adjusted for age – arguably a proxy for employment status – and the response options to questions about moderate and vigorous activity focused on leisure time physical activity. However, future investigations in these specific population subgroups may be worthwhile.

For peer review only - http://bmjopen.bmj.com/site/about/guidelines.xhtml

Strengths and limitations

Study strengths are the relatively large sample size, representativeness of the data to the American adult population ≥50 years of age, and low attrition rates. The advantages of access to and use of this dataset, however, need to be balanced against unavoidable limitations. Sleep disturbance, physical activity and pain were all assessed by self-report and are therefore susceptible to recall bias. Given the relatively broad nature of categorical response options and therefore measurement imprecision, there is also the possibility of misclassification. Our pain outcome variable specifically is broad brush (any troublesome pain, regardless of intensity or duration). However, the use of this outcome variable was based on previous research that suggests it is a good indicator of pain that interferes with daily activities[35]. Furthermore, it has been shown that pain is often reported as mild by people with chronic pain[52,53] and we would therefore have captured these cases using this single-item question.

Residual confounding cannot be excluded. We specifically chose not to take into account the consumption of medications for either sleep disturbance or pain in our analyses. From a pragmatic point of view, we were interested in problematic sleep and pain experiences – if they were being successfully managed, we considered them not to be a current problem. Finally, although prospective in design, this study only used two assessment time points. To provide greater insights into temporal processes, more intensive data collection and analysis would add value. Despite these limitations, our findings are consistent with existing evidence and, we believe, provide support for an argument to spend the time and finances required by further investigation.

For peer review only - http://bmjopen.bmj.com/site/about/guidelines.xhtml

Use of objective measures of sleep and physical activity that adhere to emerging guidelines[54] is recommended. Investigation of the possible differential impact of type of physical activity and exercise (e.g. leisure time or occupational physical activity; aerobic or strengthening exercise) may also prove illuminating. It may also be worthwhile to extend these investigations to clinical populations with pain (e.g. musculoskeletal, cancer, multiple sclerosis) to examine whether the sleep-physical activity interaction is also pertinent to the persistence or worsening of pain intensity or interference.

Conclusion

Sleep disturbance moderated the association between physical activity and incident troublesome pain in American adults aged 50 years and above. These findings warrant further examination. Replication would provide a strong case for integrating sleep health alongside physical activity recommendations for older adults to promote healthy ageing and prevention of pain onset.

Acknowledgements

We gratefully acknowledge Health and Retirement Study participants who contributed data for this study. DW is supported by a Foundation Fellowship Versus Arthritis (Award Number: 21742)

Contributions

For peer review only - http://bmjopen.bmj.com/site/about/guidelines.xhtml

DW, HMG, LMS, TJB, ALK and GLD were involved in study conception and design, and advised on the statistical analysis plan and interpretation of the data. HMG compiled the dataset and DW performed the statistical analysis. DW drafted the manuscript. HMG, LMS, TJB, ALK and GLD reviewed the manuscript, provided amendments, and approved the final version. DW, HMG and GLD had full access to study data and take responsibility for its accuracy and the integrity of the analysis.

For peer review only

Data sharing statement

Data used for this study was obtained from publically released Health and Retirement Study data products: https://hrs.isr.umich.edu/

Figure legend

Figure 1. The moderating effect of sleep disturbance on the association between physical activity and later incident pain

References

1. Shi Y, Hooten WM, Roberts RO, Warner DO. Modifiable risk factors for incidence of pain in older adults. *Pain* 2010; **151**(2): 366-71.
2. Goldberg DS, McGee SJ. Pain as a global public health priority. *BMC Public Health* 2011; **11**: 770.
3. Edwards RR. Age differences in the correlates of physical functioning in patients with chronic pain. *J Aging Health* 2006; **18**(1): 56-69.
4. Gerrits MM, van Oppen P, van Marwijk HW, Penninx BW, van der Horst HE. Pain and the onset of depressive and anxiety disorders. *Pain* 2014; **155**(1): 53-9.
5. Jakobsson U, Klevsgård R, Westergren A, Hallberg IR. Old people in pain: a comparative study. *J Pain Symptom Manage* 2003; **26**(1): 625-36.
6. Gaskin DJ, Richard P. The economic costs of pain in the United States. *J Pain* 2012; **13**(8): 715-24.
7. Patel KV, Guralnik JM, Dansie EJ, Turk DC. Prevalence and impact of pain among older adults in the United States: findings from the 2011 National Health and Aging Trends Study. *Pain* 2013; **154**(12): 2649-57.
8. United Nations DoEaSA, Population Division. World Population Ageing

2015, 2015.
9. Wilkie R, Tajar A, McBeth J. The onset of widespread musculoskeletal pain is associated with a decrease in healthy ageing in older people: a population-based prospective study. *PLoS One* 2013; **8**(3): e59858.
10. Bauman A, Merom D, Bull FC, Buchner DM, Fiatarone Singh MA. Updating the Evidence for Physical Activity: Summative Reviews of the Epidemiological Evidence, Prevalence, and Interventions to Promote "Active Aging". *Gerontologist* 2016; **56 Suppl 2**: S268-80.
11. Paterson DH, Warburton DE. Physical activity and functional limitations in older adults: a systematic review related to Canada's Physical Activity Guidelines. *Int J Behav Nutr Phys Act* 2010; **7**: 38.
12. Taylor D. Physical activity is medicine for older adults. *Postgrad Med J* 2014; **90**(1059): 26-32.
13. Galper DI, Trivedi MH, Barlow CE, Dunn AL, Kampert JB. Inverse association between physical inactivity and mental health in men and women. *Med Sci Sports Exerc* 2006; **38**(1): 173-8.
14. Sui X, Laditka JN, Church TS, et al. Prospective study of cardiorespiratory fitness and depressive symptoms in women and men. *J Psychiatr Res* 2009; **43**(5): 546-52.
15. Becofsky KM, Sui X, Lee DC, Wilcox S, Zhang J, Blair SN. A prospective study of fitness, fatness, and depressive symptoms. *Am J Epidemiol* 2015; **181**(5): 311-20.
16. Ellingson LD, Colbert LH, Cook DB. Physical activity is related to pain sensitivity in healthy women. *Med Sci Sports Exerc* 2012; **44**(7): 1401-6.
17. Landmark T, Romundstad P, Borchgrevink PC, Kaasa S, Dale O. Associations between recreational exercise and chronic pain in the general population: evidence from the HUNT 3 study. *Pain* 2011; **152**(10): 2241-7.
18. World Health Organization. World report on ageing and health.
19. Chien MY, Chen HC. Poor sleep quality is independently associated with physical disability in older adults. *J Clin Sleep Med* 2015; **11**(3): 225-32.
20. Benedict C, Byberg L, Cedernaes J, et al. Self-reported sleep disturbance is associated with Alzheimer's disease risk in men. *Alzheimers Dement* 2015; **11**(9): 1090-7.

For peer review only - http://bmjopen.bmj.com/site/about/guidelines.xhtml

21. Nagai M, Hoshide S, Kario K. Sleep duration as a risk factor for cardiovascular disease- a review of the recent literature. *Curr Cardiol Rev* 2010; **6**(1): 54-61.
22. Cappuccio FP, D'Elia L, Strazzullo P, Miller MA. Quantity and quality of sleep and incidence of type 2 diabetes: a systematic review and meta-analysis. *Diabetes Care* 2010; **33**(2): 414-20.
23. Cappuccio FP, Cooper D, D'Elia L, Strazzullo P, Miller MA. Sleep duration predicts cardiovascular outcomes: a systematic review and meta-analysis of prospective studies. *Eur Heart J* 2011; **32**(12): 1484-92.
24. Johar H, Kawan R, Emeny RT, Ladwig KH. Impaired Sleep Predicts Cognitive Decline in Old People: Findings from the Prospective KORA Age Study. *Sleep* 2016; **39**(1): 217-26.
25. Edwards RR, Almeida DM, Klick B, Haythornthwaite JA, Smith MT. Duration of sleep contributes to next-day pain report in the general population. *Pain* 2008; **137**(1): 202-7.
26. Tiede W, Magerl W, Baumgärtner U, Durrer B, Ehlert U, Treede RD. Sleep restriction attenuates amplitudes and attentional modulation of pain-related evoked potentials, but augments pain ratings in healthy volunteers. *Pain* 2010; **148**(1): 36-42.
27. Roehrs TA, Harris E, Randall S, Roth T. Pain sensitivity and recovery from mild chronic sleep loss. *Sleep* 2012; **35**(12): 1667-72.
28. Dunietz GL, Swanson LM, Jansen EC, et al. Key insomnia symptoms and incident pain in older adults: direct and mediated pathways through depression and anxiety. *Sleep* 2018; **41**(9).
29. Kline CE. The bidirectional relationship between exercise and sleep: Implications for exercise adherence and sleep improvement. *Am J Lifestyle Med* 2014; **8**(6): 375-9.
30. Lowe H, Haddock G, Mulligan LD, et al. Does exercise improve sleep for adults with insomnia? A systematic review with quality appraisal. *Clin Psychol Rev* 2019; **68**: 1-12.
31. Mesas AE, Hagen EW, Peppard PE. The bidirectional association between physical activity and sleep in middle-aged and older adults: a prospective study based on polysomnography. *Sleep* 2018; **41**(9).
32. Li J, Vitiello MV, Gooneratne NS. Sleep in Normal Aging. *Sleep Med Clin* 2018; **13**(1): 1-11.
33. Hirshkowitz M, Whiton K, Albert SM, et al. National Sleep Foundation's sleep time duration recommendations: methodology and results summary. *Sleep Health* 2015; **1**(1): 40-3.
34. Fisher GG, Ryan LH. Overview of the Health and Retirement Study and Introduction to the Special Issue. *Work Aging Retire* 2018; **4**(1): 1-9.
35. Smith D, Wilkie R, Croft P, McBeth J. Pain and Mortality in Older Adults: The Influence of Pain Phenotype. *Arthritis Care Res (Hoboken)* 2018; **70**(2): 236-43.
36. Sparling PB, Howard BJ, Dunstan DW, Owen N. Recommendations for physical activity in older adults. *BMJ* 2015; **350**: h100.
37. He XZ, Baker DW. Differences in leisure-time, household, and work-related physical activity by race, ethnicity, and education. *J Gen Intern Med* 2005; **20**(3): 259-66.
38. Tucker-Seeley RD, Subramanian SV, Li Y, Sorensen G. Neighborhood safety, socioeconomic status, and physical activity in older adults. *Am J Prev Med* 2009; **37**(3): 207-13.
39. Jenkins CD, Stanton BA, Niemcryk SJ, Rose RM. A scale for the estimation of sleep problems in clinical research. *J Clin Epidemiol* 1988; **41**(4): 313-21.
40. West B, Berglund P, Heeringa S. A closer examination of subpopulation analysis of complex-sample survey data. *Stata Journal* 2008; **8**(4): 520-31.
41. Whibley D, AlKandari N, Kristensen K, et al. Sleep and Pain: A Systematic Review of Studies of Mediation. *Clin J Pain* 2019.
42. Baglioni C, Regen W, Teghen A, et al. Sleep changes in the disorder of insomnia: a meta-analysis of polysomnographic studies. *Sleep medicine reviews* 2014; **18**(3): 195-213.

For peer review only - http://bmjopen.bmj.com/site/about/guidelines.xhtml

43. Dijk DJ. Slow-wave sleep deficiency and enhancement: implications for insomnia and its management. *World J Biol Psychiatry* 2010; **11 Suppl 1**: 22-8.
44. Krueger JM, Majde JA, Rector DM. Cytokines in immune function and sleep regulation. *Handb Clin Neurol* 2011; **98**: 229-40.
45. Besedovsky L, Lange T, Born J. Sleep and immune function. *Pflugers Arch* 2012; **463**(1): 121-37.
46. Halson SL. Sleep in elite athletes and nutritional interventions to enhance sleep. *Sports Med* 2014; **44 Suppl 1**: S13-23.
47. Reilly T, Edwards B. Altered sleep-wake cycles and physical performance in athletes. *Physiol Behav* 2007; **90**(2-3): 274-84.
48. Samuels C. Sleep, recovery, and performance: the new frontier in high-performance athletics. *Phys Med Rehabil Clin N Am* 2009; **20**(1): 149-59, ix.
49. Medic G, Wille M, Hemels ME. Short- and long-term health consequences of sleep disruption. *Nat Sci Sleep* 2017; **9**: 151-61.
50. Skarpsno ES, Mork PJ, Nilsen TIL, Jørgensen MB, Holtermann A. The joint association of musculoskeletal pain and domains of physical activity with sleep problems: cross-sectional data from the DPhacto study, Denmark. *Int Arch Occup Environ Health* 2019; **92**(4): 491-9.
51. Sluiter JK, Frings-Dresen MH, Meijman TF, van der Beek AJ. Reactivity and recovery from different types of work measured by catecholamines and cortisol: a systematic literature overview. *Occup Environ Med* 2000; **57**(5): 298-315.
52. Cassidy JD, Côté P, Carroll LJ, Kristman V. Incidence and course of low back pain episodes in the general population. *Spine (Phila Pa 1976)* 2005; **30**(24): 2817-23.
53. Dionne CE, Dunn KM, Croft PR. Does back pain prevalence really decrease with increasing age? A systematic review. *Age Ageing* 2006; **35**(3): 229-34.
54. Stamatakis E, Koster A, Hamer M, et al. Emerging collaborative research platforms for the next generation of physical activity, sleep and exercise medicine guidelines: the Prospective Physical Activity, Sitting, and Sleep consortium (ProPASS). *Br J Sports Med* 2019.

For peer review only

For peer review only - http://bmjopen.bmj.com/site/about/guidelines.xhtml

Figure 1. The moderating effect of sleep disturbance on the association between physical activity and later incident pain

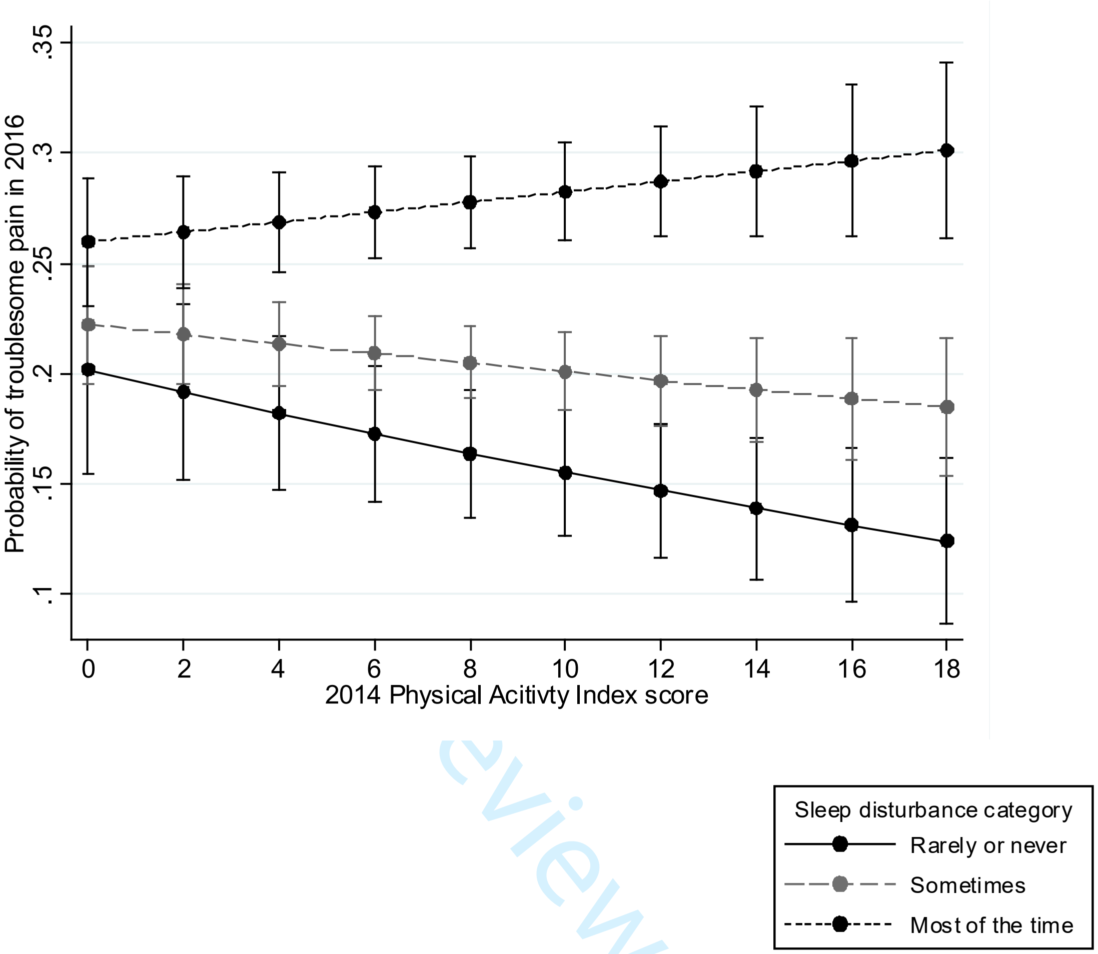

For peer review only

BMJ Open

Sleep disturbance as a moderator of the association between physical activity and later pain onset among American adults aged 50 and over: evidence from the Health and Retirement Study

Journal:	*BMJ Open*
Manuscript ID	bmjopen-2019-036219.R1
Article Type:	Original research
Date Submitted by the Author:	13-Apr-2020
Complete List of Authors:	Whibley, Daniel; University of Aberdeen, ; University of Michigan,<br>Guyer, Heidi; University of Michigan,<br>Swanson, Leslie; University of Michigan,<br>Braley, Tiffany; University of Michigan,<br>Kratz, A; University of Michigan,<br>Dunietz, Galit; University of Michigan,
**Primary Subject Heading**:	Public health
Secondary Subject Heading:	Epidemiology
Keywords:	EPIDEMIOLOGY, PUBLIC HEALTH, PAIN MANAGEMENT

SCHOLARONE™
Manuscripts

I, the Submitting Author has the right to grant and does grant on behalf of all authors of the Work (as defined in the below author licence), an exclusive licence and/or a non-exclusive licence for contributions from authors who are: i) UK Crown employees; ii) where BMJ has agreed a CC-BY licence shall apply, and/or iii) in accordance with the terms applicable for US Federal Government officers or employees acting as part of their official duties; on a worldwide, perpetual, irrevocable, royalty-free basis to BMJ Publishing Group Ltd ("BMJ") its licensees and where the relevant Journal is co-owned by BMJ to the co-owners of the Journal, to publish the Work in this journal and any other BMJ products and to exploit all rights, as set out in our licence.

The Submitting Author accepts and understands that any supply made under these terms is made by BMJ to the Submitting Author unless you are acting as an employee on behalf of your employer or a postgraduate student of an affiliated institution which is paying any applicable article publishing charge ("APC") for Open Access articles. Where the Submitting Author wishes to make the Work available on an Open Access basis (and intends to pay the relevant APC), the terms of reuse of such Open Access shall be governed by a Creative Commons licence – details of these licences and which Creative Commons licence will apply to this Work are set out in our licence referred to above.

Other than as permitted in any relevant BMJ Author's Self Archiving Policies, I confirm this Work has not been accepted for publication elsewhere, is not being considered for publication elsewhere and does not duplicate material already published. I confirm all authors consent to publication of this Work and authorise the granting of this licence.

For peer review only

Title:

Sleep disturbance as a moderator of the association between physical activity and later pain onset among American adults aged 50 and over: evidence from the Health and Retirement Study

Authors: Daniel Whibley[1,2], Heidi M. Guyer[3], Leslie M. Swanson[4], Tiffany J. Braley[5, 6], Anna L. Kratz[2], Galit Levi Dunietz[6]

Affiliations:

1. Epidemiology Group, School of Medicine, Medical Sciences and Nutrition, University of Aberdeen, Scotland, UK

2. Department of Physical Medicine and Rehabilitation, University of Michigan, Ann Arbor, MI

3. Survey Research Center. Institute for Social Research. University of Michigan, Ann Arbor, MI. RTI International, North Carolina.

4. Department of Psychiatry, University of Michigan, Ann Arbor, MI

5. Department of Neurology, Division of Multiple Sclerosis and Neuroimmunology, University of Michigan, Ann Arbor, MI

6. Department of Neurology, Division of Sleep Medicine, University of Michigan, Ann Arbor, MI

Corresponding author: Daniel Whibley, PhD, Department of Physical Medicine & Rehabilitation, University of Michigan, North Campus Research Complex, 2800 Plymouth Rd, Building NCRC B16, Ann Arbor, MI, USA; daniel.whibley@abdn.ac.uk

Keywords: Physical activity; sleep disturbance; pain; older adults

For peer review only

Abstract

Objective: To examine whether sleep disturbance modifies the association between physical activity and incident pain

Design: Prospective population-based study.

Setting: Health and Retirement Study (HRS).

Participants: American adults aged ≥50 years who reported no troublesome pain in 2014 were re-assessed for pain in 2016. Of 9828 eligible baseline respondents, 8036 (82%) had complete follow-up data for adjusted analyses (weighted analysis population N=42,407,222).

Exposures: Physical activity, assessed via interview with questions about time spent in moderate and vigorous physical activity. Sleep disturbance, assessed using a modified form of the Jenkins Sleep Scale, was examined as a potential moderator.

Main Outcome measure: Troublesome pain.

Results: In weighted analyses, 37.9% of the 2014 baseline pain-free sample participated in moderate or vigorous physical activity ≤ once a week, with an overall mean physical activity index score of 9.0 (SE 0.12). 18.6% went on to report troublesome pain in 2016. Each one-point higher on the physical activity index score was associated with a reduced odds of incident pain for those who endorsed sleep disturbance never/rarely (OR 0.97, 95%CI 0.94–0.99), but not for those who endorsed sleep disturbance sometimes (OR 0.99, 95%CI 0.97–1.01) or most of the time (OR 1.01, 95%CI 0.99–1.03). Analysis of possible interaction demonstrated that frequency of sleep disturbance moderated the physical activity-incident pain association (Wald test p=0.02).

Conclusions: The beneficial association of physical activity on reduced likelihood of later pain was only observed in persons who endorsed low levels of sleep disturbance.

For peer review only - http://bmjopen.bmj.com/site/about/guidelines.xhtml

Article summary: Strengths and limitations of this study

- This study uses data representative of the US population aged 50 years and over to provide evidence for the moderating effect of sleep disturbance on the association between level of physical activity and later pain onset.
- Strengths of the study include its complex survey design which supports population-level inference, and the low level of missing data.
- Limitations of the study include the self-reported nature of the variables under study, and the lack of available information on the presence of specific sleep disorders.

For peer review only - http://bmjopen.bmj.com/site/about/guidelines.xhtml

For peer review only

Introduction

Pain is a preeminent public health issue[1], affecting approximately 20% of the global adult population[2]. Its reach is pervasive, impacting on daily functioning[3], mental health[4], and quality of life[5]. The societal burden of pain is also vast, with annual economic costs, including those associated with healthcare and social services, exceeding $560 billion in the US alone[6]. Half of the older adult population in the US are estimated to experience troublesome pain[7]. With an increasingly older population[8] and observed associations between pain onset and a reduction in healthy ageing[9], discovery of strategies to prevent or reduce the proliferation of pain in older adults to support healthy ageing is imperative. The identification of modifiable and protective lifestyle factors that contribute to pain is key to guide population-level efforts to achieve this aim.

One such modifiable factor, and an important determinant of healthy ageing, is physical activity[10]. Physical activity is associated with lower levels of functional disability[11] and morbidity[12], better mental health[13-15], and a reduced likelihood of pain reporting[16,17]. Accordingly, physical activity is a principal health behavior included in the World Health Organization's (WHO) 2015 World Report on Ageing and Health[18].

Like low levels of physical activity, poor sleep quality and short sleep duration are associated with more functional disability[19], a wide range of diseases[20-23], poor mental health[24] and pain reporting[25-28]. Poor sleep quality and lower sleep efficiency are also associated with lower levels of physical activity and a decreased likelihood of uptake or maintenance of routine exercise[29]. Conversely, higher levels of physical activity are associated with improvements in subjectively and objectively measured sleep[30,31]. Despite this, advice about sleep behavior is rarely included in

For peer review only - http://bmjopen.bmj.com/site/about/guidelines.xhtml

physical activity guidance for older adults. A possible reason for this may be a tacit societal agreement that parameters of sleep (e.g. duration, timing and continuity) inevitably decline with age. However, although changes in sleep duration, timing and continuity are observed as humans age, most changes occur during the transition from young to middle adulthood[32]. Indeed, the National Sleep Foundation recommends that older adults sleep 7-8 hours per night (the recommendation for adults aged 18-64 is 7-9 hours)[33].

This study is predicated on the hypothesis that healthy sleep contributes to healthy ageing, and that sleep is an essential component, not only in its own right, but also as a condition within which the benefits of physical activity can be optimally reaped. To investigate the joint impact of sleep and physical activity on health, we examined whether sleep disturbance modified the association between level of physical activity and likelihood of incident troublesome pain two years later in a nationally representative sample of older adults in the US.

Methods

Study design

We used data from the Health and Retirement Study (HRS), a prospective survey of US adults aged 50 years and older conducted by the University of Michigan. Household face-to-face or telephone interviews are conducted with participants on a biannual basis across the contiguous United States[34]. Data collection is conducted by trained interviewers using laptop computers. The complex survey design of HRS incorporates multistage area probability sampling, stratification, clustering and oversampling of adults of African American and Hispanic race/ethnicity. This allows HRS to provide a nationally representative sample that, with appropriate design-based

For peer review only - http://bmjopen.bmj.com/site/about/guidelines.xhtml

analyses, can be used to make population-level inferences. A dataset comprising two HRS waves (2014 and 2016) was compiled and used to investigate relationships between physical activity, sleep disturbance, and incident pain (those with no pain in 2014 followed forward for pain status in 2016). The HRS is sponsored by the National Institute on Aging (grant number NIA U01AG009740). All data collection protocols are approved by the University of Michigan Institutional Review Board.

For peer review only

Measures

Pain

The outcome was incident troublesome pain, determined by a "yes" response to the question, "Are you often troubled with pain?" in 2016. This item has previously been shown to be comparable to pain that at least moderately interferes with daily life[35].

Physical activity

The World Health Organization (WHO) recommends that adults aged ≥65 years participate in 150 minutes of moderate physical activity or 75 minutes of vigorous physical activity each week, or an equivalent combination of both[36]. The physical health section of HRS surveys include questions about current levels of moderate and vigorous physical activity (Moderate: "how often do you take part in sports or activities that are moderately energetic such as, gardening, cleaning the car, walking at a moderate pace, dancing, floor or stretching exercises"; Vigorous: "How often do you take part in sports or activities that are vigorous, such as running or jogging, swimming, cycling, aerobics or gym workout, tennis, or digging with a spade or shovel"). Response options available for both questions are: hardly ever or never, one to three times a month, once a week, or more than once a week. Responses were used to create a physical activity index score, calculated

For peer review only - http://bmjopen.bmj.com/site/about/guidelines.xhtml

following a previously used method[37,38]. Moderate activity responses were coded as: 0= hardly ever, 1=one to three times a month, 3=once a week, 6=more than once a week. Vigorous activity responses were coded as: 0= hardly ever, 2=one to three times a month, 6=once a week, 12=more than once a week. A total physical activity index score was calculated by summing scored responses to these two questions (possible range 0–18). To provide context for physical activity index scores, a binary variable was also created, guided by WHO physical activity recommendations. Respondents were categorized as meeting the recommended level of physical activity if they participated in moderate activity more than once a week or every day, or vigorous activity more than once a week or every day. They were categorized as not meeting the recommendations if they did not participate in this amount of physical activity. According to this approach, physical activity index scores lower than 6 were indicative of physical inactivity; scores greater than 9 suggested meeting or exceeding WHO physical activity recommendations.

Sleep disturbance

Sleep disturbances assessed at baseline (2014) were investigated as a potential moderator of the physical activity (2014)-later pain reporting (2016) association. Four questions about sleep disturbance type and frequency, adapted from the Jenkins Sleep Scale[39], were included in the physical health section of HRS: How often do you have trouble falling asleep?; How often do you have trouble with waking up during the night?; How often do you have trouble with waking up too early and not being able to fall asleep again?; How often do you feel really rested when you wake up in the morning? Response options were: rarely or never; sometimes; or most of the time (reverse coded for the question about feeling rested on awakening to ensure consistency with other questions). Respondents were categorized as never or rarely experiencing sleep disturbance if they answered rarely or never to all questions; sometimes, if they answered sometimes but not

For peer review only - http://bmjopen.bmj.com/site/about/guidelines.xhtml

‘most of the time’ to any of these questions, and most of the time if they answered ‘most of the time’ to any of these questions.

Demographic and Health Characteristics

Age, gender, race/ethnicity, and number of years of school education were included as demographic covariates. Five health-related covariates were also adjusted for: Body mass index (BMI), self-reported history of depression (“Has a doctor ever told you that you have had problems with depression?”), major disease (self-reported history of cancer (any kind except skin), lung disease, heart condition, or stroke), diabetes and arthritis.

Statistical analysis

Analyses were conducted using Stata (version 15.1, StataCorp LLC, College Station, TX). HRS sample weights were used to correct for unequal probability of selection that may have arisen as a result of the multistage sampling design. Demographic and health-related characteristics were summarized (mean, standard error). To investigate whether sleep disturbance reported in 2014 moderated the relationship between level of physical activity at this time and incident pain in 2016, logistic regression was performed with a physical activity index-sleep disturbance status interaction term. As the research question focused on those with no troublesome pain at baseline, subpopulation estimation was used to compute point and variance estimates[40]. This ensured that those reporting no pain at baseline contributed data to the calculation of effect estimates while those with and without pain at baseline contributed data to calculation of standard errors. In the presence of significant interaction ($p<0.05$), predicted probabilities of reporting pain at follow-up were calculated and plotted according to physical activity index score and sleep disturbance status. Models were adjusted for age, gender, race/ethnicity, number of years of school education,

For peer review only - http://bmjopen.bmj.com/site/about/guidelines.xhtml

BMI, self-reported history of depression, major disease (self-reported history of cancer (any kind except skin), lung disease, heart condition, or stroke), diabetes and arthritis.

Patient involvement

Patients were not involved in this study and there are no plans to disseminate the findings to HRS participants.

Results

Descriptive analysis

Of 16,384 HRS respondents, 16,330 (99.7%) answered the question about troublesome pain in 2014. Of these, 9,828 (60.2%) reported not being troubled by pain. Baseline demographic and health-related characteristics of this pain-free sample are presented in Table 1. After applying sample weights, 51.5% were female, and the mean age was 66.9.

Demographic and health-related characteristics and incident pain

Data on reported pain status was provided by 8,513 (86.6%) respondents in 2016. After applying survey weights, an estimated 18.6% (SE 0.005) of the sampled population had self-reported incident troublesome pain in 2016. This was associated with female gender, older age, higher BMI, less education, being separated, divorced or widowed, poorer self-rated health, having a history of depression, major disease, Alzheimer's Disease or dementia, arthritis, diabetes, a higher frequency of sleep disturbance, and lower levels of physical activity (Table 2). Having missing data for pain status in 2016 was associated with older age, higher BMI, Hispanic ethnicity, less formal

For peer review only - http://bmjopen.bmj.com/site/about/guidelines.xhtml

education, being widowed, poorer self-rated health, a history of major disease, Alzheimer's disease or dementia, and lower physical activity levels in 2014.

Physical activity

37.9% (SE 0.008) of the baseline population participated in moderate or vigorous physical activity once a week or less. Overall, the mean baseline physical activity index score was 9.0 (SE 0.12). For those participating in moderate or vigorous physical activity once a week or less, the mean physical activity index score was 2.5 (SE 0.07); for those who reported participating in moderate or vigorous activity more than once a week or every day, the mean physical activity index score was 12.9 (SE 0.10).

Sleep

22.3% (SE 0.006) of the baseline pain-free sample reported rarely or never having sleep disturbance, 45.9% (SE 0.007) reported sometimes experiencing sleep disturbance, and 31.8% (SE 0.005) reported experiencing sleep disturbance most of the time.

The association between physical activity and incident troublesome pain and the moderating effect of sleep disturbance

In models adjusted for pre-specified covariates and stratified by sleep disturbance categories, each one point higher on the physical activity index score (range 0–18) was associated with a reduced likelihood of troublesome pain onset for those who reported "never/rarely" having sleep disturbance (OR 0.97, 95%CI 0.94–0.99). The effect attenuated with increasing frequency of sleep disturbance (sleep disturbance sometimes: OR 0.99, 95%CI 0.97–1.01; sleep disturbance most of the time: OR 1.01, 95%CI 0.99–1.03). In an adjusted model that included a physical activity index

For peer review only - http://bmjopen.bmj.com/site/about/guidelines.xhtml

score-frequency of sleep disturbance interaction term, a significant moderating effect of sleep disturbance on the physical activity index score-incident pain association was observed (Wald test for the interaction: p=0.02) (Table 3, Figure 1).

Table 1: Demographic and health characteristics of Health and Retirement Study (HRS) respondents not troubled by pain in 2014

	Unweighted frequency (N)[a]	Weighted proportion or mean (SE)[a]
Gender		
Female	5346	51.5% (0.005)
Male	4482	48.5% (0.005)
Age (mean, SE)	9828	66.9 (0.3)
BMI		
Underweight/normal weight	2816	31.2% (0.006)
Overweight	3426	38.9% (0.007)
Obese	1820	20.1% (0.006)
Obese, BMI ≥35	920	9.9% (0.004)
Missing data	846 (8.6%)	
Race/ethnicity		
White	6371	78.7% (0.01)
Black	1837	9.6% (0.006)
Hispanic	1301	8.4% (0.01)
Other	319	3.4% (0.003)
Years of school (mean, SE)	9792	13.5 (0.08)
Missing data	36 (0.4%)	
Marital status in 2014		
Married	5759	62.6% (0.007)
Separated or divorced	1644	16.2% (0.005)
Widowed	1943	14.4% (0.004)
Never married	481	6.8% (0.004)
Missing data	1 (0.01%)	
Self-rated health		
Excellent	1131	14.0% (0.005)
Very good	3611	40.6% (0.008)
Good	3255	30.7% (0.007)
Fair	1499	12.0% (0.006)
Poor	323	2.5% (0.002)
Don't know/refused	9 (0.09%)	
History of depression		
Yes	1554	16.8% (0.005)
No	8203	83.2% (0.005)
Missing data	71 (0.7%)	
History of major disease[†]		

For peer review only - http://bmjopen.bmj.com/site/about/guidelines.xhtml

Yes	3766	35.5% (0.008)
No	5960	64.5% (0.008)
Missing data	102 (1.0%)	
Alzheimer's Disease or dementia		
Yes	274	2.0% (0.001)
No	9510	98.0% (0.001)
Missing data	44 (0.5%)	
Arthritis		
Yes	4653	43.8% (0.008)
No	5175	56.2% (0.008)
Diabetes		
Yes	2273	19.9% (0.005)
No	7486	80.1% (0.005)
Missing data	69 (0.7%)	
Sleep disturbance		
Rarely or never	2131	22.3% (0.006)
Sometimes	4404	45.9% (0.007)
Most of the time	3253	31.8% (0.005)
Missing data	40 (0.4%)	
Physical activity		
Moderate or vigorous >1 week	5668	62.1% (0.008)
Moderate or vigorous ≤1 week	4151	37.9% (0.008
Missing data	9 (0.09%)	
Physical activity index score	9763	9.0 (0.12)
Missing data	65 (0.7%)	

[a] Unweighted frequencies represent sample counts; weighted proportion or mean (linearized SE) represent corresponding estimated population values (through sampling weights, 9,828 represent 50,923,453 community-dwelling American adults aged 50 years and above).

† Major disease defined as having a history of cancer (excluding skin), lung disease, heart condition, or stroke.

For peer review only - http://bmjopen.bmj.com/site/about/guidelines.xhtml

Table 2. Physical activity index scores in 2014 and incident pain in 2016 by demographic and health-related factors

	2014 Physical Activity Index		2016 Incident pain	
	Weighted mean or Beta, (SE)	p value	Weighted proportion or Odds Ratio, (SE)	p value
Gender				
Female	8.3 (0.1)	<0.001	20.1% (0.008)	
Male	9.6 (0.2)		16.9% (0.008)	0.01
Age	B = -0.2 (0.009)	<0.001	OR = 1.01 (0.004)	0.03
BMI				
Underweight/normal weight	9.9 (0.2)		15.4 (0.008)	
Overweight	9.5 (0.2)		16.8 (0.008)	
Obese	8.4 (0.2)		22.5 (0.01)	
Obese, BMI ≥35	6.4 (0.2)	<0.001	26.7 (0.02)	<0.001
Race/ethnicity				
White	9.2 (0.1)		17.9 (0.006)	
Black	7.7 (0.2)		19.4 (0.01)	
Hispanic	8.4 (0.4)		23.6 (0.02)	
Other	8.7 (0.5)	<0.001	19.4 (0.03)	0.06
Years of school	B = 0.5 (0.04)	<0.001	OR = 0.9 (0.008)	<0.001
Marital status				
Married	9.6 (0.2)		17.5% (0.007)	
Separated or divorced	9.0 (0.2)		21.2% (SE 0.01)	
Widowed	6.7 (0.2)		21.6% (0.01)	
Never married	7.9 (0.3)	<0.001	16.5% (0.03)	0.002
Self-rated health				
Excellent	12.3 (0.3)		9.0% (0.01)	
Very good	9.9 (0.2)		14.9% (0.006)	
Good	7.8 (0.1)		22.9% (0.01)	
Fair	6.0 (0.3)		30.2% (0.02)	
Poor	2.6 (0.2)	<0.001	34.7% (0.03)	<0.001
History of depression				
Yes	7.9 (0.3)		29.3% (0.01)	
No	9.2 (0.1)	<0.001	16.3% (0.006)	<0.001
History of major disease†				
Yes	7.5 (0.1)		22.8% (0.009)	
No	9.8 (0.2)	<0.001	16.4% (0.007)	<0.001
Alzheimer's Disease or dementia				
Yes	4.1 (0.5)		30.7% (0.03)	
No	9.1 (0.1)	<0.001	18.4% (0.005)	<0.001
Arthritis				
Yes	8.2 (0.1)		26.8% (0.007)	
No	9.5 (0.2)	<0.001	12.3% (0.006)	<0.001
Diabetes				
Yes	7.1 (0.2)		23.1% (0.01)	

For peer review only - http://bmjopen.bmj.com/site/about/guidelines.xhtml

No	9.4 (0.1)	<0.001	17.5% (0.005)	<0.001
Sleep disturbance				
Rarely or never	9.9 (0.2)		11.8% (0.01)	
Sometimes	9.0 (0.2)		17.0% (0.007)	
Most of the time	8.3 (0.2)	<0.001	25.7% (0.01)	<0.001
2014 Physical Activity Index				
Moderate or vigorous >1 week			17.2% (0.006)	
Moderate or vigorous ≤1 week			21.0% (0.008)	<0.001
Physical activity index score			OR = 0.97 (0.004)	<0.001

† Major disease defined as having a history of cancer (excluding skin), lung disease, heart condition, or stroke.

For peer review only - http://bmjopen.bmj.com/site/about/guidelines.xhtml

Table 3. Logistic regression analysis investigating the moderating effect of sleep disturbance category on the association between 2014 physical activity index score and likelihood of troublesome pain in 2016*

	Odds Ratio (95% CI)	Linearized SE	T	P
**Physical activity index score** (PAI)	0.97 (0.94–0.99)	0.01	-2.46	0.02
**Sleep disturbance category**				
Rarely or never	Reference			
Sometimes	1.14 (0.78–1.67)	0.22	0.68	0.50
Most of the time	1.42 (0.99–2.05)	0.26	1.92	0.06
**Physical activity index score * sleep disturbance category interaction term**				
Rarely or never	Reference			
Sometimes	1.02 (0.99–1.06)	0.02	1.18	0.24[a]
Most of the time	1.05 (1.01–1.08)	0.02	2.79	0.01[a]
**Age**	0.99 (0.99–1.00)	0.004	-1.57	0.12
**Gender**				
Male	Reference			
Female	1.02 (0.85–1.23)	0.10	0.20	0.84
**BMI**				
Underweight/normal weight	Reference			
Overweight	1.05 (0.88–1.26)	0.09	0.57	0.57
Obese	1.36 (1.09–1.70)	0.15	2.74	0.01
Obese, BMI ≥35	1.56 (1.22–1.99)	0.19	3.63	0.001
**Race/ethnicity**				
White	Reference			
Black	1.02 (0.85–1.22)	0.09	0.22	0.83
Hispanic	1.27 (0.92–1.77)	0.21	1.49	0.14
Other	1.28 (0.86–1.89)	0.25	1.25	0.22
**Years of school**	0.95 (0.93–0.97)	0.01	-5.13	<0.001
**History of depression**	1.69 (1.38–2.07)	0.17	5.17	<0.001
**History of major disease**†	1.24 (1.02–1.51)	0.12	2.22	0.03
**Arthritis**	2.35 (2.00–2.78)	0.19	10.41	<0.001
**Diabetes**	1.05 (0.92–1.21)	0.07	0.73	0.47

* Adjusted analysis N=8,036

CI: confidence interval; SE: standard error

[a] Wald test statistic for overall interaction: p=0.02

† Major disease defined as having a history of cancer (excluding skin), lung disease, heart condition, or stroke.

For peer review only - http://bmjopen.bmj.com/site/about/guidelines.xhtml

Discussion

This nationally representative prospective cohort of US adults shows that self-reported sleep disturbance moderates the association between physical activity and later pain. Although higher levels of physical activity were associated with reduced likelihood of incident troublesome pain among those who reported sleep disturbance never, rarely, or sometimes, the benefit of higher levels of physical activity were not observed for those who reported disturbed sleep most of the time.

Previous cross-sectional research has demonstrated linear, negative associations between increasing levels of exercise frequency, duration and intensity and the presence of chronic pain[17]. Our finding that increasing levels of physical activity are associated with a reduced likelihood of later pain reporting is consistent with this and, in addition to the prospective nature of our study, we have extended the evidence by identifying a moderating effect of sleep disturbance on this association. Given our results, it is possible that the beneficial influence of physical activity on pain reporting has previously been underestimated, and perhaps muted by a proportion having unmeasured or unaccounted for chronic sleep disturbance.

Mechanisms by which sleep disturbance may impact the association between level of physical activity and later pain onset are likely multidimensional[41], potentially acting as both moderators and mediators. Sleep disturbance, including delayed sleep onset, waking up during the night, and waking up earlier than intended, can be associated with reduced total sleep time and, consequently, less time spent in non-rapid eye movement slow-wave sleep (SWS)[42]. Adequate

For peer review only - http://bmjopen.bmj.com/site/about/guidelines.xhtml

For peer review only

SWS is thought to contribute to the recuperative function of sleep. A reduced duration of SWS and therefore protracted restorative sleep, has been shown to reduce next-day performance[43] and may diminish the beneficial effects of physical activity by limiting recovery. Suboptimal sleep also has a negative impact on immune function[44,45], and a body of evidence supports a case for sleep as essential to recovery after exercise in elite athletes[46-48]. It is plausible that similar mechanisms may be at play for older adults who participate in higher levels of physical activity – within the context of disturbed sleep the benefit of higher levels of physical activity may not only be weakened, but may render sleep-disturbed individuals more susceptible to poor health outcomes.

Focusing exclusively on a working population, Skarpsno and colleagues examined the role of both occupational and leisure time physical activity on later sleep disturbance[49]. For those with pain, higher levels of objectively measured physical activity (both at work and during leisure time) were associated with a higher prevalence of sleep disturbance. Skarpsno and colleagues argue that higher levels of occupational physical activity may lead to an overload response[49] (as described by Sluiter and colleagues[50]), increasing the probability of sleep disturbance. We hypothesize that such an overload response may also be pertinent to our observation of the negative influence of sleep disturbance on the physical activity-pain relationship. In the absence of adequate restorative sleep, benefits of physical activity may be counterpointed by an aggregating overload response. However, with a population aged 50 years and above, the motivation driving different modes of physical activity may be salient; retirees who choose to participate in higher levels of physical activity may have very different experiences to those over 50 in employment, particularly those in physically demanding jobs.

For peer review only - http://bmjopen.bmj.com/site/about/guidelines.xhtml

We have investigated any sleep disturbance as a moderator of the physical activity-pain onset association. However, given that each individual sleep item could be argued as representing an independent sleep disturbance domain with different implications on pain reporting, in post-hoc analysis we explored the potentially moderating effect of each specific sleep disturbance (trouble falling asleep, staying asleep, waking too early, and feeling unrefreshed upon awakening). The moderating effect was most pronounced for trouble falling asleep, and all sleep disturbance domains exhibited a similar pattern of effect, with the exception that waking too early showed no sign of moderating the physical activity-pain onset association. Focused research into the moderating effect of specific sleep domains, including those not available in the HRS dataset (e.g. sleep duration) are recommended in future research where more granular data collection, including objective assessments, could help to comprehensively address questions about the impact of specific sleep domains on the physical activity-pain onset association.

Strengths and limitations

Study strengths are the relatively large sample size, representativeness of the data to the American adult population ≥50 years of age, and low attrition rates. The advantages of access to and use of this dataset, however, need to be balanced against unavoidable limitations. Sleep disturbance, physical activity and pain were all assessed by self-report and are therefore susceptible to recall bias. Given the broad nature of response categories for pain and sleep disturbances, measurement imprecision may have resulted in misclassification that could have biased the effect estimates. The HRS questionnaire uses different response categories to the Jenkins Sleep Scale and, in the absence of comparative data, the validity of these items remains open to question. Future validation study of HRS sleep item responses against the Jenkins Sleep

For peer review only - http://bmjopen.bmj.com/site/about/guidelines.xhtml

Scale would benefit future sleep-related research that uses the HRS dataset. Our pain outcome variable specifically is broad brush (any troublesome pain, regardless of intensity or duration). However, the use of this outcome variable was based on previous research that suggests it is a good indicator of pain that interferes with daily activities[35]. Furthermore, it has been shown that pain is often reported as mild by people with chronic pain[51,52] and we would therefore have captured these cases using this single-item question.

Residual confounding cannot be excluded. We specifically chose not to take into account the consumption of medications for either sleep disturbance or pain in our analyses. From a pragmatic point of view, we were interested in problematic sleep and pain experiences – if they were being successfully managed, we considered them not to be a current problem. Also, although we adjusted for a history of depression, we did not have information on whether participants were taking antidepressant medications. Given potential effects on both sleep and pain reporting, collecting this information and including it in future analyses is recommended. An additional source of potential confounding given the age of the cohort is the built environment and living arrangements. In a post-hoc sensitivity analysis we adjusted for nursing home status and living arrangements and the interpretation of our findings remained unchanged. Finally, although prospective in design, this study only used two assessment time points. To provide greater insights into temporal processes, more intensive data collection and analysis would add value. Despite these limitations, our findings are consistent with existing evidence and, we believe, provide support for an argument to spend the time and finances required by further investigation.

For peer review only - http://bmjopen.bmj.com/site/about/guidelines.xhtml

Use of objective measures of sleep and physical activity that adhere to emerging guidelines[53] is recommended. Investigation of the possible differential impact of type of physical activity and exercise (e.g. leisure time or occupational physical activity; aerobic or strengthening exercise) may also prove illuminating. It may also be worthwhile to extend these investigations to clinical populations with pain (e.g. musculoskeletal, cancer, multiple sclerosis) to examine whether the sleep-physical activity interaction is also pertinent to the persistence or worsening of pain intensity or interference.

Conclusion

Sleep disturbance moderated the association between physical activity and incident troublesome pain in American adults aged 50 years and above. These findings warrant further examination. Replication would provide a strong case for integrating sleep health alongside physical activity recommendations for older adults to promote healthy ageing and prevention of pain onset.

Acknowledgements

We gratefully acknowledge Health and Retirement Study participants who contributed data for this study.

Contributor statement

DW, HMG, LMS, TJB, ALK and GLD were involved in study conception and design, and advised on the statistical analysis plan and interpretation of the data. HMG compiled the dataset and DW performed the statistical analysis. DW drafted the manuscript. HMG, LMS, TJB, ALK and GLD

For peer review only - http://bmjopen.bmj.com/site/about/guidelines.xhtml

For peer review only

reviewed the manuscript, provided amendments, and approved the final version. DW, HMG and GLD had full access to study data and take responsibility for its accuracy and the integrity of the analysis.

Competing interest

There are no competing interests for any author

Funding

DW is supported by a Foundation Fellowship Versus Arthritis (Award Number: 21742)

Data sharing statement

Data are available in a public, open access repository. The dataset used for this study was generated from data products publicly released by the Health and Retirement Study (HRS): https://hrs.isr.umich.edu. The HRS is sponsored by the National Institute on Aging (grant number NIA U01AG009740).

Figure legend

Figure 1. The moderating effect of sleep disturbance on the association between physical activity and later incident pain

For peer review only

References

1. Shi Y, Hooten WM, Roberts RO, Warner DO. Modifiable risk factors for incidence of pain in older adults. *Pain* 2010; **151**(2): 366-71.
2. Goldberg DS, McGee SJ. Pain as a global public health priority. *BMC Public Health* 2011; **11**: 770.
3. Edwards RR. Age differences in the correlates of physical functioning in patients with chronic pain. *J Aging Health* 2006; **18**(1): 56-69.
4. Gerrits MM, van Oppen P, van Marwijk HW, Penninx BW, van der Horst HE. Pain and the onset of depressive and anxiety disorders. *Pain* 2014; **155**(1): 53-9.
5. Jakobsson U, Klevsgård R, Westergren A, Hallberg IR. Old people in pain: a comparative study. *J Pain Symptom Manage* 2003; **26**(1): 625-36.
6. Gaskin DJ, Richard P. The economic costs of pain in the United States. *J Pain* 2012; **13**(8): 715-24.
7. Patel KV, Guralnik JM, Dansie EJ, Turk DC. Prevalence and impact of pain among older adults in the United States: findings from the 2011 National Health and Aging Trends Study. *Pain* 2013; **154**(12): 2649-57.
8. United Nations DoEaSA, Population Division. World Population Ageing

2015, 2015.
9. Wilkie R, Tajar A, McBeth J. The onset of widespread musculoskeletal pain is associated with a decrease in healthy ageing in older people: a population-based prospective study. *PLoS One* 2013; **8**(3): e59858.
10. Bauman A, Merom D, Bull FC, Buchner DM, Fiatarone Singh MA. Updating the Evidence for Physical Activity: Summative Reviews of the Epidemiological Evidence, Prevalence, and Interventions to Promote "Active Aging". *Gerontologist* 2016; **56 Suppl 2**: S268-80.
11. Paterson DH, Warburton DE. Physical activity and functional limitations in older adults: a systematic review related to Canada's Physical Activity Guidelines. *Int J Behav Nutr Phys Act* 2010; **7**: 38.
12. Taylor D. Physical activity is medicine for older adults. *Postgrad Med J* 2014; **90**(1059): 26-32.
13. Galper DI, Trivedi MH, Barlow CE, Dunn AL, Kampert JB. Inverse association between physical inactivity and mental health in men and women. *Med Sci Sports Exerc* 2006; **38**(1): 173-8.
14. Sui X, Laditka JN, Church TS, et al. Prospective study of cardiorespiratory fitness and depressive symptoms in women and men. *J Psychiatr Res* 2009; **43**(5): 546-52.
15. Becofsky KM, Sui X, Lee DC, Wilcox S, Zhang J, Blair SN. A prospective study of fitness, fatness, and depressive symptoms. *Am J Epidemiol* 2015; **181**(5): 311-20.
16. Ellingson LD, Colbert LH, Cook DB. Physical activity is related to pain sensitivity in healthy women. *Med Sci Sports Exerc* 2012; **44**(7): 1401-6.
17. Landmark T, Romundstad P, Borchgrevink PC, Kaasa S, Dale O. Associations between recreational exercise and chronic pain in the general population: evidence from the HUNT 3 study. *Pain* 2011; **152**(10): 2241-7.
18. World Health Organization. World report on ageing and health.
19. Chien MY, Chen HC. Poor sleep quality is independently associated with physical disability in older adults. *J Clin Sleep Med* 2015; **11**(3): 225-32.
20. Benedict C, Byberg L, Cedernaes J, et al. Self-reported sleep disturbance is associated with Alzheimer's disease risk in men. *Alzheimers Dement* 2015; **11**(9): 1090-7.

For peer review only - http://bmjopen.bmj.com/site/about/guidelines.xhtml

21. Nagai M, Hoshide S, Kario K. Sleep duration as a risk factor for cardiovascular disease- a review of the recent literature. *Curr Cardiol Rev* 2010; **6**(1): 54-61.
22. Cappuccio FP, D'Elia L, Strazzullo P, Miller MA. Quantity and quality of sleep and incidence of type 2 diabetes: a systematic review and meta-analysis. *Diabetes Care* 2010; **33**(2): 414-20.
23. Cappuccio FP, Cooper D, D'Elia L, Strazzullo P, Miller MA. Sleep duration predicts cardiovascular outcomes: a systematic review and meta-analysis of prospective studies. *Eur Heart J* 2011; **32**(12): 1484-92.
24. Johar H, Kawan R, Emeny RT, Ladwig KH. Impaired Sleep Predicts Cognitive Decline in Old People: Findings from the Prospective KORA Age Study. *Sleep* 2016; **39**(1): 217-26.
25. Edwards RR, Almeida DM, Klick B, Haythornthwaite JA, Smith MT. Duration of sleep contributes to next-day pain report in the general population. *Pain* 2008; **137**(1): 202-7.
26. Tiede W, Magerl W, Baumgärtner U, Durrer B, Ehlert U, Treede RD. Sleep restriction attenuates amplitudes and attentional modulation of pain-related evoked potentials, but augments pain ratings in healthy volunteers. *Pain* 2010; **148**(1): 36-42.
27. Roehrs TA, Harris E, Randall S, Roth T. Pain sensitivity and recovery from mild chronic sleep loss. *Sleep* 2012; **35**(12): 1667-72.
28. Dunietz GL, Swanson LM, Jansen EC, et al. Key insomnia symptoms and incident pain in older adults: direct and mediated pathways through depression and anxiety. *Sleep* 2018; **41**(9).
29. Kline CE. The bidirectional relationship between exercise and sleep: Implications for exercise adherence and sleep improvement. *Am J Lifestyle Med* 2014; **8**(6): 375-9.
30. Lowe H, Haddock G, Mulligan LD, et al. Does exercise improve sleep for adults with insomnia? A systematic review with quality appraisal. *Clin Psychol Rev* 2019; **68**: 1-12.
31. Mesas AE, Hagen EW, Peppard PE. The bidirectional association between physical activity and sleep in middle-aged and older adults: a prospective study based on polysomnography. *Sleep* 2018; **41**(9).
32. Li J, Vitiello MV, Gooneratne NS. Sleep in Normal Aging. *Sleep Med Clin* 2018; **13**(1): 1-11.
33. Hirshkowitz M, Whiton K, Albert SM, et al. National Sleep Foundation's sleep time duration recommendations: methodology and results summary. *Sleep Health* 2015; **1**(1): 40-3.
34. Fisher GG, Ryan LH. Overview of the Health and Retirement Study and Introduction to the Special Issue. *Work Aging Retire* 2018; **4**(1): 1-9.
35. Smith D, Wilkie R, Croft P, McBeth J. Pain and Mortality in Older Adults: The Influence of Pain Phenotype. *Arthritis Care Res (Hoboken)* 2018; **70**(2): 236-43.
36. Sparling PB, Howard BJ, Dunstan DW, Owen N. Recommendations for physical activity in older adults. *BMJ* 2015; **350**: h100.
37. He XZ, Baker DW. Differences in leisure-time, household, and work-related physical activity by race, ethnicity, and education. *J Gen Intern Med* 2005; **20**(3): 259-66.
38. Tucker-Seeley RD, Subramanian SV, Li Y, Sorensen G. Neighborhood safety, socioeconomic status, and physical activity in older adults. *Am J Prev Med* 2009; **37**(3): 207-13.
39. Jenkins CD, Stanton BA, Niemcryk SJ, Rose RM. A scale for the estimation of sleep problems in clinical research. *J Clin Epidemiol* 1988; **41**(4): 313-21.
40. West B, Berglund P, Heeringa S. A closer examination of subpopulation analysis of complex-sample survey data. *Stata Journal* 2008; **8**(4): 520-31.
41. Whibley D, AlKandari N, Kristensen K, et al. Sleep and Pain: A Systematic Review of Studies of Mediation. *Clin J Pain* 2019.
42. Baglioni C, Regen W, Teghen A, et al. Sleep changes in the disorder of insomnia: a meta-analysis of polysomnographic studies. *Sleep medicine reviews* 2014; **18**(3): 195-213.

For peer review only - http://bmjopen.bmj.com/site/about/guidelines.xhtml

43. Dijk DJ. Slow-wave sleep deficiency and enhancement: implications for insomnia and its management. *World J Biol Psychiatry* 2010; **11 Suppl 1**: 22-8.
44. Krueger JM, Majde JA, Rector DM. Cytokines in immune function and sleep regulation. *Handb Clin Neurol* 2011; **98**: 229-40.
45. Besedovsky L, Lange T, Born J. Sleep and immune function. *Pflugers Arch* 2012; **463**(1): 121-37.
46. Halson SL. Sleep in elite athletes and nutritional interventions to enhance sleep. *Sports Med* 2014; **44 Suppl 1**: S13-23.
47. Reilly T, Edwards B. Altered sleep-wake cycles and physical performance in athletes. *Physiol Behav* 2007; **90**(2-3): 274-84.
48. Samuels C. Sleep, recovery, and performance: the new frontier in high-performance athletics. *Phys Med Rehabil Clin N Am* 2009; **20**(1): 149-59, ix.
49. Skarpsno ES, Mork PJ, Nilsen TIL, Jørgensen MB, Holtermann A. The joint association of musculoskeletal pain and domains of physical activity with sleep problems: cross-sectional data from the DPhacto study, Denmark. *Int Arch Occup Environ Health* 2019; **92**(4): 491-9.
50. Sluiter JK, Frings-Dresen MH, Meijman TF, van der Beek AJ. Reactivity and recovery from different types of work measured by catecholamines and cortisol: a systematic literature overview. *Occup Environ Med* 2000; **57**(5): 298-315.
51. Cassidy JD, Côté P, Carroll LJ, Kristman V. Incidence and course of low back pain episodes in the general population. *Spine (Phila Pa 1976)* 2005; **30**(24): 2817-23.
52. Dionne CE, Dunn KM, Croft PR. Does back pain prevalence really decrease with increasing age? A systematic review. *Age Ageing* 2006; **35**(3): 229-34.
53. Stamatakis E, Koster A, Hamer M, et al. Emerging collaborative research platforms for the next generation of physical activity, sleep and exercise medicine guidelines: the Prospective Physical Activity, Sitting, and Sleep consortium (ProPASS). *Br J Sports Med* 2019.

For peer review only

For peer review only - http://bmjopen.bmj.com/site/about/guidelines.xhtml

Figure 1. The moderating effect of sleep disturbance on the association between physical activity and later incident pain

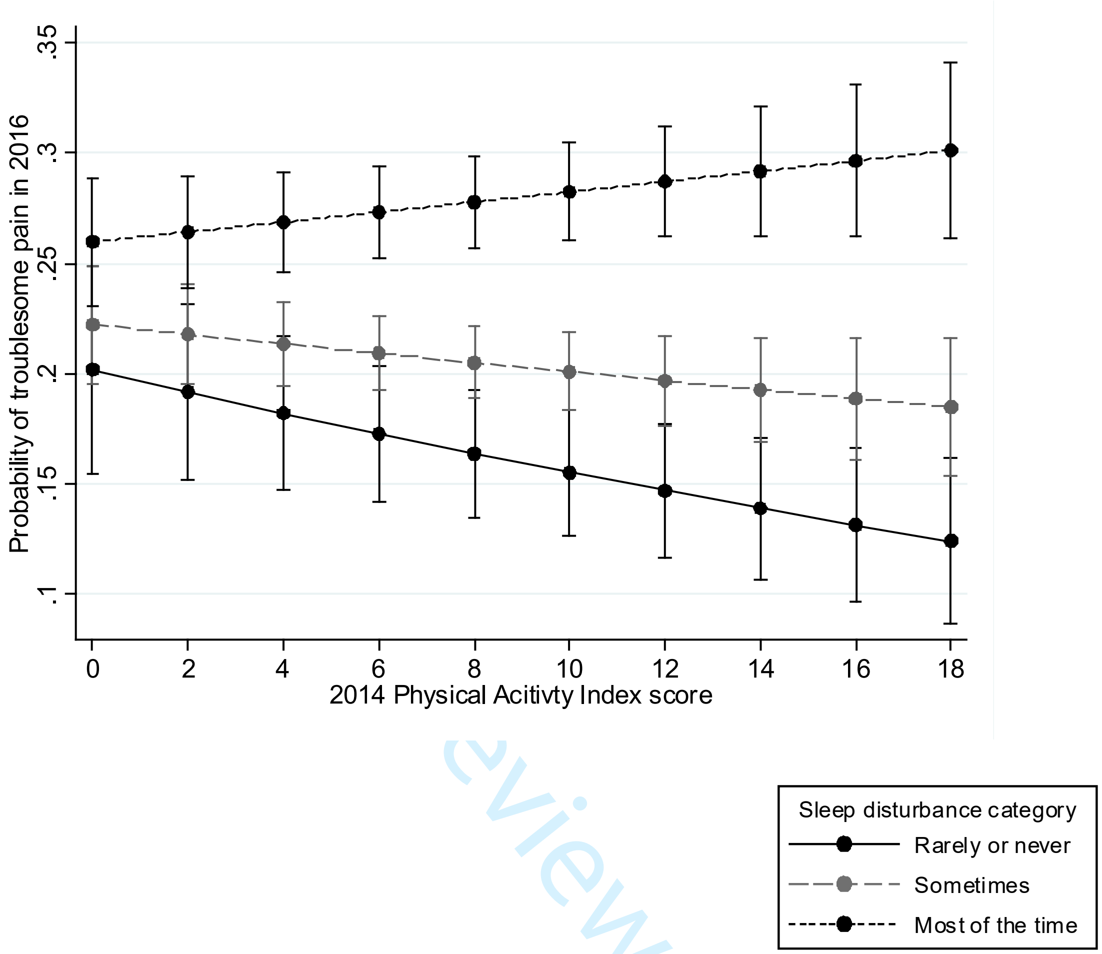

STROBE Statement—Checklist of items that should be included in reports of ***cohort studies***

	Item No	Recommendation	Page No
**Title and abstract**	1	(*a*) Indicate the study's design with a commonly used term in the title or the abstract<br>(*b*) Provide in the abstract an informative and balanced summary of what was done and what was found	2
**Introduction**			
Background/rationale	2	Explain the scientific background and rationale for the investigation being reported	4-5
Objectives	3	State specific objectives, including any prespecified hypotheses	5
**Methods**			
Study design	4	Present key elements of study design early in the paper	5-9
Setting	5	Describe the setting, locations, and relevant dates, including periods of recruitment, exposure, follow-up, and data collection	5-6
Participants	6	(*a*) Give the eligibility criteria, and the sources and methods of selection of participants. Describe methods of follow-up<br>(*b*) For matched studies, give matching criteria and number of exposed and unexposed	5-6
Variables	7	Clearly define all outcomes, exposures, predictors, potential confounders, and effect modifiers. Give diagnostic criteria, if applicable	6-8
Data sources/ measurement	8*	For each variable of interest, give sources of data and details of methods of assessment (measurement). Describe comparability of assessment methods if there is more than one group	5-8
Bias	9	Describe any efforts to address potential sources of bias	8-9
Study size	10	Explain how the study size was arrived at	9
Quantitative variables	11	Explain how quantitative variables were handled in the analyses. If applicable, describe which groupings were chosen and why	6-9
Statistical methods	12	(*a*) Describe all statistical methods, including those used to control for confounding<br>(*b*) Describe any methods used to examine subgroups and interactions<br>(*c*) Explain how missing data were addressed<br>(*d*) If applicable, explain how loss to follow-up was addressed<br>(*e*) Describe any sensitivity analyses	8-9
**Results**			
Participants	13*	(a) Report numbers of individuals at each stage of study—eg numbers potentially eligible, examined for eligibility, confirmed eligible, included in the study, completing follow-up, and analysed<br>(b) Give reasons for non-participation at each stage<br>(c) Consider use of a flow diagram	9
Descriptive data	14*	(a) Give characteristics of study participants (eg demographic, clinical, social) and information on exposures and potential confounders<br>(b) Indicate number of participants with missing data for each variable of interest<br>(c) Summarise follow-up time (eg, average and total amount)	9-10
Outcome data	15*	Report numbers of outcome events or summary measures over time	9-10; 13-14

For peer review only

For peer review only - http://bmjopen.bmj.com/site/about/guidelines.xhtml

Main results	16	(*a*) Give unadjusted estimates and, if applicable, confounder-adjusted estimates and their precision (eg, 95% confidence interval). Make clear which confounders were adjusted for and why they were included<br>(*b*) Report category boundaries when continuous variables were categorized<br>(*c*) If relevant, consider translating estimates of relative risk into absolute risk for a meaningful time period	10-11; 13-15
Other analyses	17	Report other analyses done—eg analyses of subgroups and interactions, and sensitivity analyses	10-11; 15; 18; 19
**Discussion**			
Key results	18	Summarise key results with reference to study objectives	16
Limitations	19	Discuss limitations of the study, taking into account sources of potential bias or imprecision. Discuss both direction and magnitude of any potential bias	18-20
Interpretation	20	Give a cautious overall interpretation of results considering objectives, limitations, multiplicity of analyses, results from similar studies, and other relevant evidence	20
Generalisability	21	Discuss the generalisability (external validity) of the study results	18
**Other information**			
Funding	22	Give the source of funding and the role of the funders for the present study and, if applicable, for the original study on which the present article is based	6;20

*Give information separately for exposed and unexposed groups.

For peer review only

Sleep disturbance as a moderator of the association between physical activity and later pain onset among American adults aged 50 and over: evidence from the Health and Retirement Study

Journal:	*BMJ Open*
Manuscript ID	bmjopen-2019-036219.R2
Article Type:	Original research
Date Submitted by the Author:	02-May-2020
Complete List of Authors:	Whibley, Daniel; University of Aberdeen, ; University of Michigan,<br>Guyer, Heidi; University of Michigan,<br>Swanson, Leslie; University of Michigan,<br>Braley, Tiffany; University of Michigan,<br>Kratz, A; University of Michigan,<br>Dunietz, Galit; University of Michigan,
**Primary Subject Heading**:	Public health
Secondary Subject Heading:	Epidemiology
Keywords:	EPIDEMIOLOGY, PUBLIC HEALTH, PAIN MANAGEMENT

SCHOLARONE™
Manuscripts

I, the Submitting Author has the right to grant and does grant on behalf of all authors of the Work (as defined in the below author licence), an exclusive licence and/or a non-exclusive licence for contributions from authors who are: i) UK Crown employees; ii) where BMJ has agreed a CC-BY licence shall apply, and/or iii) in accordance with the terms applicable for US Federal Government officers or employees acting as part of their official duties; on a worldwide, perpetual, irrevocable, royalty-free basis to BMJ Publishing Group Ltd ("BMJ") its licensees and where the relevant Journal is co-owned by BMJ to the co-owners of the Journal, to publish the Work in this journal and any other BMJ products and to exploit all rights, as set out in our licence.

The Submitting Author accepts and understands that any supply made under these terms is made by BMJ to the Submitting Author unless you are acting as an employee on behalf of your employer or a postgraduate student of an affiliated institution which is paying any applicable article publishing charge ("APC") for Open Access articles. Where the Submitting Author wishes to make the Work available on an Open Access basis (and intends to pay the relevant APC), the terms of reuse of such Open Access shall be governed by a Creative Commons licence – details of these licences and which Creative Commons licence will apply to this Work are set out in our licence referred to above.

Other than as permitted in any relevant BMJ Author's Self Archiving Policies, I confirm this Work has not been accepted for publication elsewhere, is not being considered for publication elsewhere and does not duplicate material already published. I confirm all authors consent to publication of this Work and authorise the granting of this licence.

For peer review only

Title:

Sleep disturbance as a moderator of the association between physical activity and later pain onset among American adults aged 50 and over: evidence from the Health and Retirement Study

Authors: Daniel Whibley[1,2], Heidi M. Guyer[3], Leslie M. Swanson[4], Tiffany J. Braley[5, 6], Anna L. Kratz[2], Galit Levi Dunietz[6]

Affiliations:

1. Epidemiology Group, School of Medicine, Medical Sciences and Nutrition, University of Aberdeen, Scotland, UK

2. Department of Physical Medicine and Rehabilitation, University of Michigan, Ann Arbor, MI

3. Survey Research Center. Institute for Social Research. University of Michigan, Ann Arbor, MI. RTI International, North Carolina.

4. Department of Psychiatry, University of Michigan, Ann Arbor, MI

5. Department of Neurology, Division of Multiple Sclerosis and Neuroimmunology, University of Michigan, Ann Arbor, MI

6. Department of Neurology, Division of Sleep Medicine, University of Michigan, Ann Arbor, MI

Corresponding author: Daniel Whibley, PhD, Department of Physical Medicine & Rehabilitation, University of Michigan, North Campus Research Complex, 2800 Plymouth Rd, Building NCRC B16, Ann Arbor, MI, USA; daniel.whibley@abdn.ac.uk

Keywords: Physical activity; sleep disturbance; pain; older adults

For peer review only - http://bmjopen.bmj.com/site/about/guidelines.xhtml

Abstract

Objective: To examine whether sleep disturbance modifies the association between physical activity and incident pain

Design: Prospective population-based study.

Setting: Health and Retirement Study (HRS).

Participants: American adults aged ≥50 years who reported no troublesome pain in 2014 were re-assessed for pain in 2016. Of 9828 eligible baseline respondents, 8036 (82%) had complete follow-up data for adjusted analyses (weighted analysis population N=42,407,222).

Exposures: Physical activity, assessed via interview with questions about time spent in moderate and vigorous physical activity. Sleep disturbance, assessed using a modified form of the Jenkins Sleep Scale, was examined as a potential moderator.

Main Outcome measure: Troublesome pain.

Results: In weighted analyses, 37.9% of the 2014 baseline pain-free sample participated in moderate or vigorous physical activity ≤ once a week, with an overall mean physical activity index score of 9.0 (SE 0.12). 18.6% went on to report troublesome pain in 2016. Each one-point higher on the physical activity index score was associated with a reduced odds of incident pain for those who endorsed sleep disturbance never/rarely (OR 0.97, 95%CI 0.94–0.99), but not for those who endorsed sleep disturbance sometimes (OR 0.99, 95%CI 0.97–1.01) or most of the time (OR 1.01, 95%CI 0.99–1.03). Analysis of possible interaction demonstrated that frequency of sleep disturbance moderated the physical activity-incident pain association (Wald test p=0.02).

Conclusions: The beneficial association of physical activity on reduced likelihood of later pain was only observed in persons who endorsed low levels of sleep disturbance.

For peer review only - http://bmjopen.bmj.com/site/about/guidelines.xhtml

Article summary: Strengths and limitations of this study

- This study uses data representative of the US population aged 50 years and over to provide evidence for the moderating effect of sleep disturbance on the association between level of physical activity and later pain onset.
- Strengths of the study include its complex survey design which supports population-level inference, and the low level of missing data.
- Limitations of the study include the self-reported nature of the variables under study, and the lack of available information on the presence of specific sleep disorders.

For peer review only

For peer review only - http://bmjopen.bmj.com/site/about/guidelines.xhtml

Introduction

Pain is a preeminent public health issue[1], affecting approximately 20% of the global adult population[2]. Its reach is pervasive, impacting on daily functioning[3], mental health[4], and quality of life[5]. The societal burden of pain is also vast, with annual economic costs, including those associated with healthcare and social services, exceeding $560 billion in the US alone[6]. Half of the older adult population in the US are estimated to experience troublesome pain[7]. With an increasingly older population[8] and observed associations between pain onset and a reduction in healthy ageing[9], discovery of strategies to prevent or reduce the proliferation of pain in older adults to support healthy ageing is imperative. The identification of modifiable and protective lifestyle factors that contribute to pain is key to guide population-level efforts to achieve this aim.

One such modifiable factor, and an important determinant of healthy ageing, is physical activity[10]. Physical activity is associated with lower levels of functional disability[11] and morbidity[12], better mental health[13-15], and a reduced likelihood of pain reporting[16,17]. Accordingly, physical activity is a principal health behavior included in the World Health Organization's (WHO) 2015 World Report on Ageing and Health[18].

Like low levels of physical activity, poor sleep quality and short sleep duration are associated with more functional disability[19], a wide range of diseases[20-23], poor mental health[24] and pain reporting[25-28]. Poor sleep quality and lower sleep efficiency are also associated with lower levels of physical activity and a decreased likelihood of uptake or maintenance of routine exercise[29]. Conversely, higher levels of physical activity are associated with improvements in subjectively and objectively measured sleep[30,31]. Despite this, advice about sleep behavior is rarely included in

For peer review only - http://bmjopen.bmj.com/site/about/guidelines.xhtml

physical activity guidance for older adults. A possible reason for this may be a tacit societal agreement that parameters of sleep (e.g. duration, timing and continuity) inevitably decline with age. However, although changes in sleep duration, timing and continuity are observed as humans age, most changes occur during the transition from young to middle adulthood[32]. Indeed, the National Sleep Foundation recommends that older adults sleep 7-8 hours per night (the recommendation for adults aged 18-64 is 7-9 hours)[33].

This study is predicated on the hypothesis that healthy sleep contributes to healthy ageing, and that sleep is an essential component, not only in its own right, but also as a condition within which the benefits of physical activity can be optimally reaped. To investigate the joint impact of sleep and physical activity on health, we examined whether sleep disturbance modified the association between level of physical activity and likelihood of incident troublesome pain two years later in a nationally representative sample of older adults in the US.

Methods

Study design

We used data from the Health and Retirement Study (HRS), a prospective survey of US adults aged 50 years and older conducted by the University of Michigan. Household face-to-face or telephone interviews are conducted with participants on a biannual basis across the contiguous United States[34]. Data collection is conducted by trained interviewers using laptop computers. The complex survey design of HRS incorporates multistage area probability sampling, stratification, clustering and oversampling of adults of African American and Hispanic race/ethnicity. This allows HRS to provide a nationally representative sample that, with appropriate design-based

For peer review only - http://bmjopen.bmj.com/site/about/guidelines.xhtml

analyses, can be used to make population-level inferences. A dataset comprising two HRS waves (2014 and 2016) was compiled and used to investigate relationships between physical activity, sleep disturbance, and incident pain (those with no pain in 2014 followed forward for pain status in 2016). The HRS is sponsored by the National Institute on Aging (grant number NIA U01AG009740). All data collection protocols are approved by the University of Michigan Institutional Review Board.

Measures

Pain

The outcome was incident troublesome pain, determined by a "yes" response to the question, "Are you often troubled with pain?" in 2016. This item has previously been shown to be comparable to pain that at least moderately interferes with daily life[35].

Physical activity

The World Health Organization (WHO) recommends that adults aged ≥65 years participate in 150 minutes of moderate physical activity or 75 minutes of vigorous physical activity each week, or an equivalent combination of both[36]. The physical health section of HRS surveys include questions about current levels of moderate and vigorous physical activity (Moderate: "how often do you take part in sports or activities that are moderately energetic such as, gardening, cleaning the car, walking at a moderate pace, dancing, floor or stretching exercises"; Vigorous: "How often do you take part in sports or activities that are vigorous, such as running or jogging, swimming, cycling, aerobics or gym workout, tennis, or digging with a spade or shovel"). Response options available for both questions are: hardly ever or never, one to three times a month, once a week, or more than once a week. Responses were used to create a physical activity index score, calculated

For peer review only - http://bmjopen.bmj.com/site/about/guidelines.xhtml

following a previously used method[37,38]. Moderate activity responses were coded as: 0= hardly ever, 1=one to three times a month, 3=once a week, 6=more than once a week. Vigorous activity responses were coded as: 0= hardly ever, 2=one to three times a month, 6=once a week, 12=more than once a week. A total physical activity index score was calculated by summing scored responses to these two questions (possible range 0–18). To provide context for physical activity index scores, a binary variable was also created, guided by WHO physical activity recommendations. Respondents were categorized as meeting the recommended level of physical activity if they participated in moderate activity more than once a week or every day, or vigorous activity more than once a week or every day. They were categorized as not meeting the recommendations if they did not participate in this amount of physical activity. According to this approach, physical activity index scores lower than 6 were indicative of physical inactivity; scores greater than 9 suggested meeting or exceeding WHO physical activity recommendations.

Sleep disturbance

Sleep disturbances assessed at baseline (2014) were investigated as a potential moderator of the physical activity (2014)-later pain reporting (2016) association. Four questions about sleep disturbance type and frequency, adapted from the Jenkins Sleep Scale[39], were included in the physical health section of HRS: How often do you have trouble falling asleep?; How often do you have trouble with waking up during the night?; How often do you have trouble with waking up too early and not being able to fall asleep again?; How often do you feel really rested when you wake up in the morning? Response options were: rarely or never; sometimes; or most of the time (reverse coded for the question about feeling rested on awakening to ensure consistency with other questions). Respondents were categorized as never or rarely experiencing sleep disturbance if they answered rarely or never to all questions; sometimes, if they answered sometimes but not

For peer review only - http://bmjopen.bmj.com/site/about/guidelines.xhtml

For peer review only

'most of the time' to any of these questions, and most of the time if they answered 'most of the time' to any of these questions.

Demographic and Health Characteristics

Age, gender, race/ethnicity, and number of years of school education were included as demographic covariates. Five health-related covariates were also adjusted for: Body mass index (BMI), self-reported history of depression ("Has a doctor ever told you that you have had problems with depression?"), major disease (self-reported history of cancer (any kind except skin), lung disease, heart condition, or stroke), diabetes and arthritis.

Statistical analysis

Analyses were conducted using Stata (version 15.1, StataCorp LLC, College Station, TX). HRS sample weights were used to correct for unequal probability of selection that may have arisen as a result of the multistage sampling design. Demographic and health-related characteristics were summarized (mean, standard error). To investigate whether sleep disturbance reported in 2014 moderated the relationship between level of physical activity at this time and incident pain in 2016, logistic regression was performed with a physical activity index-sleep disturbance status interaction term. As the research question focused on those with no troublesome pain at baseline, subpopulation estimation was used to compute point and variance estimates[40]. This ensured that those reporting no pain at baseline contributed data to the calculation of effect estimates while those with and without pain at baseline contributed data to calculation of standard errors. In the presence of significant interaction ($p<0.05$), predicted probabilities of reporting pain at follow-up were calculated and plotted according to physical activity index score and sleep disturbance status. Models were adjusted for age, gender, race/ethnicity, number of years of school education,

For peer review only - http://bmjopen.bmj.com/site/about/guidelines.xhtml

BMI, self-reported history of depression, major disease (self-reported history of cancer (any kind except skin), lung disease, heart condition, or stroke), diabetes and arthritis.

Patient involvement

Patients were not involved in this study and there are no plans to disseminate the findings to HRS participants.

Results

Descriptive analysis

Of 16,384 HRS respondents, 16,330 (99.7%) answered the question about troublesome pain in 2014. Of these, 9,828 (60.2%) reported not being troubled by pain. Baseline demographic and health-related characteristics of this pain-free sample are presented in Table 1. After applying sample weights, 51.5% were female, and the mean age was 66.9.

Demographic and health-related characteristics and incident pain

Data on reported pain status was provided by 8,513 (86.6%) respondents in 2016. After applying survey weights, an estimated 18.6% (SE 0.005) of the sampled population had self-reported incident troublesome pain in 2016. This was associated with female gender, older age, higher BMI, less education, being separated, divorced or widowed, poorer self-rated health, having a history of depression, major disease, Alzheimer's Disease or dementia, arthritis, diabetes, a higher frequency of sleep disturbance, and lower levels of physical activity (Table 2). Having missing data for pain status in 2016 was associated with older age, higher BMI, Hispanic ethnicity, less formal

For peer review only - http://bmjopen.bmj.com/site/about/guidelines.xhtml

education, being widowed, poorer self-rated health, a history of major disease, Alzheimer's disease or dementia, and lower physical activity levels in 2014.

Physical activity

37.9% (SE 0.008) of the baseline population participated in moderate or vigorous physical activity once a week or less. Overall, the mean baseline physical activity index score was 9.0 (SE 0.12). For those participating in moderate or vigorous physical activity once a week or less, the mean physical activity index score was 2.5 (SE 0.07); for those who reported participating in moderate or vigorous activity more than once a week or every day, the mean physical activity index score was 12.9 (SE 0.10).

Sleep

22.3% (SE 0.006) of the baseline pain-free sample reported rarely or never having sleep disturbance, 45.9% (SE 0.007) reported sometimes experiencing sleep disturbance, and 31.8% (SE 0.005) reported experiencing sleep disturbance most of the time.

The association between physical activity and incident troublesome pain and the moderating effect of sleep disturbance

In models adjusted for pre-specified covariates and stratified by sleep disturbance categories, each one point higher on the physical activity index score (range 0–18) was associated with a reduced likelihood of troublesome pain onset for those who reported "never/rarely" having sleep disturbance (OR 0.97, 95%CI 0.94–0.99). The effect attenuated with increasing frequency of sleep disturbance (sleep disturbance sometimes: OR 0.99, 95%CI 0.97–1.01; sleep disturbance most of the time: OR 1.01, 95%CI 0.99–1.03). In an adjusted model that included a physical activity index

For peer review only - http://bmjopen.bmj.com/site/about/guidelines.xhtml

score-frequency of sleep disturbance interaction term, a significant moderating effect of sleep disturbance on the physical activity index score-incident pain association was observed (Wald test for the interaction: p=0.02) (Table 3, Figure 1).

Table 1: Demographic and health characteristics of Health and Retirement Study (HRS) respondents not troubled by pain in 2014

	Unweighted frequency (N)[a]	Weighted proportion or mean (SE)[a]
Gender		
Female	5346	51.5% (0.005)
Male	4482	48.5% (0.005)
Age (mean, SE)	9828	66.9 (0.3)
BMI		
Underweight/normal weight	2816	31.2% (0.006)
Overweight	3426	38.9% (0.007)
Obese	1820	20.1% (0.006)
Obese, BMI ≥35	920	9.9% (0.004)
Missing data	846 (8.6%)	
Race/ethnicity		
White	6371	78.7% (0.01)
Black	1837	9.6% (0.006)
Hispanic	1301	8.4% (0.01)
Other	319	3.4% (0.003)
Years of school (mean, SE)	9792	13.5 (0.08)
Missing data	36 (0.4%)	
Marital status in 2014		
Married	5759	62.6% (0.007)
Separated or divorced	1644	16.2% (0.005)
Widowed	1943	14.4% (0.004)
Never married	481	6.8% (0.004)
Missing data	1 (0.01%)	
Self-rated health		
Excellent	1131	14.0% (0.005)
Very good	3611	40.6% (0.008)
Good	3255	30.7% (0.007)
Fair	1499	12.0% (0.006)
Poor	323	2.5% (0.002)
Don't know/refused	9 (0.09%)	
History of depression		
Yes	1554	16.8% (0.005)
No	8203	83.2% (0.005)
Missing data	71 (0.7%)	
History of major disease[†]		

For peer review only - http://bmjopen.bmj.com/site/about/guidelines.xhtml

Yes	3766	35.5% (0.008)
No	5960	64.5% (0.008)
Missing data	102 (1.0%)	
Alzheimer's Disease or dementia		
Yes	274	2.0% (0.001)
No	9510	98.0% (0.001)
Missing data	44 (0.5%)	
Arthritis		
Yes	4653	43.8% (0.008)
No	5175	56.2% (0.008)
Diabetes		
Yes	2273	19.9% (0.005)
No	7486	80.1% (0.005)
Missing data	69 (0.7%)	
Sleep disturbance		
Rarely or never	2131	22.3% (0.006)
Sometimes	4404	45.9% (0.007)
Most of the time	3253	31.8% (0.005)
Missing data	40 (0.4%)	
Physical activity		
Moderate or vigorous >1 week	5668	62.1% (0.008)
Moderate or vigorous ≤1 week	4151	37.9% (0.008
Missing data	9 (0.09%)	
Physical activity index score	9763	9.0 (0.12)
Missing data	65 (0.7%)	

[a] Unweighted frequencies represent sample counts; weighted proportion or mean (linearized SE) represent corresponding estimated population values (through sampling weights, 9,828 represent 50,923,453 community-dwelling American adults aged 50 years and above).

† Major disease defined as having a history of cancer (excluding skin), lung disease, heart condition, or stroke.

For peer review only - http://bmjopen.bmj.com/site/about/guidelines.xhtml

Table 2. Physical activity index scores in 2014 and incident pain in 2016 by demographic and health-related factors

	2014 Physical Activity Index		2016 Incident pain	
	Weighted mean or Beta, (SE)	p value	Weighted proportion or Odds Ratio, (SE)	p value
Gender				
Female	8.3 (0.1)	<0.001	20.1% (0.008)	
Male	9.6 (0.2)		16.9% (0.008)	0.01
Age	B = -0.2 (0.009)	<0.001	OR = 1.01 (0.004)	0.03
BMI				
Underweight/normal weight	9.9 (0.2)		15.4 (0.008)	
Overweight	9.5 (0.2)		16.8 (0.008)	
Obese	8.4 (0.2)		22.5 (0.01)	
Obese, BMI ≥35	6.4 (0.2)	<0.001	26.7 (0.02)	<0.001
Race/ethnicity				
White	9.2 (0.1)		17.9 (0.006)	
Black	7.7 (0.2)		19.4 (0.01)	
Hispanic	8.4 (0.4)		23.6 (0.02)	
Other	8.7 (0.5)	<0.001	19.4 (0.03)	0.06
Years of school	B = 0.5 (0.04)	<0.001	OR = 0.9 (0.008)	<0.001
Marital status				
Married	9.6 (0.2)		17.5% (0.007)	
Separated or divorced	9.0 (0.2)		21.2% (SE 0.01)	
Widowed	6.7 (0.2)		21.6% (0.01)	
Never married	7.9 (0.3)	<0.001	16.5% (0.03)	0.002
Self-rated health				
Excellent	12.3 (0.3)		9.0% (0.01)	
Very good	9.9 (0.2)		14.9% (0.006)	
Good	7.8 (0.1)		22.9% (0.01)	
Fair	6.0 (0.3)		30.2% (0.02)	
Poor	2.6 (0.2)	<0.001	34.7% (0.03)	<0.001
History of depression				
Yes	7.9 (0.3)		29.3% (0.01)	
No	9.2 (0.1)	<0.001	16.3% (0.006)	<0.001
History of major disease†				
Yes	7.5 (0.1)		22.8% (0.009)	
No	9.8 (0.2)	<0.001	16.4% (0.007)	<0.001
Alzheimer's Disease or dementia				
Yes	4.1 (0.5)		30.7% (0.03)	
No	9.1 (0.1)	<0.001	18.4% (0.005)	<0.001
Arthritis				
Yes	8.2 (0.1)		26.8% (0.007)	
No	9.5 (0.2)	<0.001	12.3% (0.006)	<0.001
Diabetes				
Yes	7.1 (0.2)		23.1% (0.01)	

For peer review only - http://bmjopen.bmj.com/site/about/guidelines.xhtml

No	9.4 (0.1)	<0.001	17.5% (0.005)	<0.001
Sleep disturbance				
Rarely or never	9.9 (0.2)		11.8% (0.01)	
Sometimes	9.0 (0.2)		17.0% (0.007)	
Most of the time	8.3 (0.2)	<0.001	25.7% (0.01)	<0.001
2014 Physical Activity Index				
Moderate or vigorous >1 week			17.2% (0.006)	
Moderate or vigorous ≤1 week			21.0% (0.008)	<0.001
Physical activity index score			OR = 0.97 (0.004)	<0.001

† Major disease defined as having a history of cancer (excluding skin), lung disease, heart condition, or stroke.

For peer review only

Table 3. Logistic regression analysis investigating the moderating effect of sleep disturbance category on the association between 2014 physical activity index score and likelihood of troublesome pain in 2016*

	Odds Ratio (95% CI)	Linearized SE	T	P
**Physical activity index score** (PAI)	0.97 (0.94–0.99)	0.01	-2.46	0.02
**Sleep disturbance category**				
Rarely or never	Reference			
Sometimes	1.14 (0.78–1.67)	0.22	0.68	0.50
Most of the time	1.42 (0.99–2.05)	0.26	1.92	0.06
**Physical activity index score * sleep disturbance category interaction term**				
Rarely or never	Reference			
Sometimes	1.02 (0.99–1.06)	0.02	1.18	0.24[a]
Most of the time	1.05 (1.01–1.08)	0.02	2.79	0.01[a]
**Age**	0.99 (0.99–1.00)	0.004	-1.57	0.12
**Gender**				
Male	Reference			
Female	1.02 (0.85–1.23)	0.10	0.20	0.84
**BMI**				
Underweight/normal weight	Reference			
Overweight	1.05 (0.88–1.26)	0.09	0.57	0.57
Obese	1.36 (1.09–1.70)	0.15	2.74	0.01
Obese, BMI ≥35	1.56 (1.22–1.99)	0.19	3.63	0.001
**Race/ethnicity**				
White	Reference			
Black	1.02 (0.85–1.22)	0.09	0.22	0.83
Hispanic	1.27 (0.92–1.77)	0.21	1.49	0.14
Other	1.28 (0.86–1.89)	0.25	1.25	0.22
**Years of school**	0.95 (0.93–0.97)	0.01	-5.13	<0.001
**History of depression**	1.69 (1.38–2.07)	0.17	5.17	<0.001
**History of major disease**[†]	1.24 (1.02–1.51)	0.12	2.22	0.03
**Arthritis**	2.35 (2.00–2.78)	0.19	10.41	<0.001
**Diabetes**	1.05 (0.92–1.21)	0.07	0.73	0.47

* Adjusted analysis N=8,036

CI: confidence interval; SE: standard error

[a] Wald test statistic for overall interaction: p=0.02

[†] Major disease defined as having a history of cancer (excluding skin), lung disease, heart condition, or stroke.

For peer review only - http://bmjopen.bmj.com/site/about/guidelines.xhtml

Discussion

This nationally representative prospective cohort of US adults shows that self-reported sleep disturbance moderates the association between physical activity and later pain. Although higher levels of physical activity were associated with reduced likelihood of incident troublesome pain among those who reported sleep disturbance never, rarely, or sometimes, the benefit of higher levels of physical activity were not observed for those who reported disturbed sleep most of the time.

Previous cross-sectional research has demonstrated linear, negative associations between increasing levels of exercise frequency, duration and intensity and the presence of chronic pain[17]. Our finding that increasing levels of physical activity are associated with a reduced likelihood of later pain reporting is consistent with this and, in addition to the prospective nature of our study, we have extended the evidence by identifying a moderating effect of sleep disturbance on this association. Given our results, it is possible that the beneficial influence of physical activity on pain reporting has previously been underestimated, and perhaps muted by a proportion having unmeasured or unaccounted for chronic sleep disturbance.

Mechanisms by which sleep disturbance may impact the association between level of physical activity and later pain onset are likely multidimensional[41], potentially acting as both moderators and mediators. Sleep disturbance, including delayed sleep onset, waking up during the night, and waking up earlier than intended, can be associated with reduced total sleep time and, consequently, less time spent in non-rapid eye movement slow-wave sleep (SWS)[42]. Adequate

For peer review only - http://bmjopen.bmj.com/site/about/guidelines.xhtml

SWS is thought to contribute to the recuperative function of sleep. A reduced duration of SWS and therefore protracted restorative sleep, has been shown to reduce next-day performance[43] and may diminish the beneficial effects of physical activity by limiting recovery. Suboptimal sleep also has a negative impact on immune function[44,45], and a body of evidence supports a case for sleep as essential to recovery after exercise in elite athletes[46-48]. It is plausible that similar mechanisms may be at play for older adults who participate in higher levels of physical activity – within the context of disturbed sleep the benefit of higher levels of physical activity may not only be weakened, but may render sleep-disturbed individuals more susceptible to poor health outcomes.

Focusing exclusively on a working population, Skarpsno and colleagues examined the role of both occupational and leisure time physical activity on later sleep disturbance[49]. For those with pain, higher levels of objectively measured physical activity (both at work and during leisure time) were associated with a higher prevalence of sleep disturbance. Skarpsno and colleagues argue that higher levels of occupational physical activity may lead to an overload response[49] (as described by Sluiter and colleagues[50]), increasing the probability of sleep disturbance. We hypothesize that such an overload response may also be pertinent to our observation of the negative influence of sleep disturbance on the physical activity-pain relationship. In the absence of adequate restorative sleep, benefits of physical activity may be counterpointed by an aggregating overload response. However, with a population aged 50 years and above, the motivation driving different modes of physical activity may be salient; retirees who choose to participate in higher levels of physical activity may have very different experiences to those over 50 in employment, particularly those in physically demanding jobs.

For peer review only - http://bmjopen.bmj.com/site/about/guidelines.xhtml

We have investigated any sleep disturbance as a moderator of the physical activity-pain onset association. However, given that each individual sleep item could be argued as representing an independent sleep disturbance domain with different implications on pain reporting, in post-hoc analysis we explored the potentially moderating effect of each specific sleep disturbance (trouble falling asleep, staying asleep, waking too early, and feeling unrefreshed upon awakening). The moderating effect was most pronounced for trouble falling asleep, and all sleep disturbance domains exhibited a similar pattern of effect, with the exception that waking too early showed no sign of moderating the physical activity-pain onset association (Supplement 1). Focused research into the moderating effect of specific sleep domains, including those not available in the HRS dataset (e.g. sleep duration) are recommended in future research where more granular data collection, including objective assessments, could help to comprehensively address questions about the impact of specific sleep domains on the physical activity-pain onset association.

Strengths and limitations

Study strengths are the relatively large sample size, representativeness of the data to the American adult population ≥50 years of age, and low attrition rates. The advantages of access to and use of this dataset, however, need to be balanced against unavoidable limitations. Sleep disturbance, physical activity and pain were all assessed by self-report and are therefore susceptible to recall bias. Given the broad nature of response categories for pain and sleep disturbances, measurement imprecision may have resulted in misclassification that could have biased the effect estimates. The HRS questionnaire uses different response categories to the Jenkins Sleep Scale and, in the absence of comparative data, the validity of these items remains open to question. Future validation study of HRS sleep item responses against the Jenkins Sleep

For peer review only - http://bmjopen.bmj.com/site/about/guidelines.xhtml

Scale would benefit future sleep-related research that uses the HRS dataset. Our pain outcome variable specifically is broad brush (any troublesome pain, regardless of intensity or duration). However, the use of this outcome variable was based on previous research that suggests it is a good indicator of pain that interferes with daily activities[35]. Furthermore, it has been shown that pain is often reported as mild by people with chronic pain[51,52] and we would therefore have captured these cases using this single-item question.

Residual confounding cannot be excluded. We specifically chose not to take into account the consumption of medications for either sleep disturbance or pain in our analyses. From a pragmatic point of view, we were interested in problematic sleep and pain experiences – if they were being successfully managed, we considered them not to be a current problem. Also, although we adjusted for a history of depression, we did not have information on whether participants were taking antidepressant medications. Given potential effects on both sleep and pain reporting, collecting this information and including it in future analyses is recommended. An additional source of potential confounding given the age of the cohort is the built environment and living arrangements. In a post-hoc sensitivity analysis we adjusted for nursing home status and living arrangements and the interpretation of our findings remained unchanged (Supplement 2). Finally, although prospective in design, this study only used two assessment time points. To provide greater insights into temporal processes, more intensive data collection and analysis would add value. Despite these limitations, our findings are consistent with existing evidence and, we believe, provide support for an argument to spend the time and finances required by further investigation.

For peer review only - http://bmjopen.bmj.com/site/about/guidelines.xhtml

Use of objective measures of sleep and physical activity that adhere to emerging guidelines[53] is recommended. Investigation of the possible differential impact of type of physical activity and exercise (e.g. leisure time or occupational physical activity; aerobic or strengthening exercise) may also prove illuminating. It may also be worthwhile to extend these investigations to clinical populations with pain (e.g. musculoskeletal, cancer, multiple sclerosis) to examine whether the sleep-physical activity interaction is also pertinent to the persistence or worsening of pain intensity or interference.

For peer review only

Conclusion

Sleep disturbance moderated the association between physical activity and incident troublesome pain in American adults aged 50 years and above. These findings warrant further examination. Replication would provide a strong case for integrating sleep health alongside physical activity recommendations for older adults to promote healthy ageing and prevention of pain onset.

Acknowledgements

We gratefully acknowledge Health and Retirement Study participants who contributed data for this study.

Contributor statement

DW, HMG, LMS, TJB, ALK and GLD were involved in study conception and design, and advised on the statistical analysis plan and interpretation of the data. HMG compiled the dataset and DW performed the statistical analysis. DW drafted the manuscript. HMG, LMS, TJB, ALK and GLD

For peer review only - http://bmjopen.bmj.com/site/about/guidelines.xhtml

reviewed the manuscript, provided amendments, and approved the final version. DW, HMG and GLD had full access to study data and take responsibility for its accuracy and the integrity of the analysis.

Competing interest

There are no competing interests for any author

Funding

DW is supported by a Foundation Fellowship Versus Arthritis (Award Number: 21742)

Data sharing statement

Data are available in a public, open access repository. The dataset used for this study was generated from data products publicly released by the Health and Retirement Study (HRS): https://hrs.isr.umich.edu. The HRS is sponsored by the National Institute on Aging (grant number NIA U01AG009740).

Figure legend

Figure 1. The moderating effect of sleep disturbance on the association between physical activity and later incident pain

For peer review only - http://bmjopen.bmj.com/site/about/guidelines.xhtml

References

1. Shi Y, Hooten WM, Roberts RO, Warner DO. Modifiable risk factors for incidence of pain in older adults. *Pain* 2010; **151**(2): 366-71.
2. Goldberg DS, McGee SJ. Pain as a global public health priority. *BMC Public Health* 2011; **11**: 770.
3. Edwards RR. Age differences in the correlates of physical functioning in patients with chronic pain. *J Aging Health* 2006; **18**(1): 56-69.
4. Gerrits MM, van Oppen P, van Marwijk HW, Penninx BW, van der Horst HE. Pain and the onset of depressive and anxiety disorders. *Pain* 2014; **155**(1): 53-9.
5. Jakobsson U, Klevsgård R, Westergren A, Hallberg IR. Old people in pain: a comparative study. *J Pain Symptom Manage* 2003; **26**(1): 625-36.
6. Gaskin DJ, Richard P. The economic costs of pain in the United States. *J Pain* 2012; **13**(8): 715-24.
7. Patel KV, Guralnik JM, Dansie EJ, Turk DC. Prevalence and impact of pain among older adults in the United States: findings from the 2011 National Health and Aging Trends Study. *Pain* 2013; **154**(12): 2649-57.
8. United Nations DoEaSA, Population Division. World Population Ageing

2015, 2015.
9. Wilkie R, Tajar A, McBeth J. The onset of widespread musculoskeletal pain is associated with a decrease in healthy ageing in older people: a population-based prospective study. *PLoS One* 2013; **8**(3): e59858.
10. Bauman A, Merom D, Bull FC, Buchner DM, Fiatarone Singh MA. Updating the Evidence for Physical Activity: Summative Reviews of the Epidemiological Evidence, Prevalence, and Interventions to Promote "Active Aging". *Gerontologist* 2016; **56 Suppl 2**: S268-80.
11. Paterson DH, Warburton DE. Physical activity and functional limitations in older adults: a systematic review related to Canada's Physical Activity Guidelines. *Int J Behav Nutr Phys Act* 2010; **7**: 38.
12. Taylor D. Physical activity is medicine for older adults. *Postgrad Med J* 2014; **90**(1059): 26-32.
13. Galper DI, Trivedi MH, Barlow CE, Dunn AL, Kampert JB. Inverse association between physical inactivity and mental health in men and women. *Med Sci Sports Exerc* 2006; **38**(1): 173-8.
14. Sui X, Laditka JN, Church TS, et al. Prospective study of cardiorespiratory fitness and depressive symptoms in women and men. *J Psychiatr Res* 2009; **43**(5): 546-52.
15. Becofsky KM, Sui X, Lee DC, Wilcox S, Zhang J, Blair SN. A prospective study of fitness, fatness, and depressive symptoms. *Am J Epidemiol* 2015; **181**(5): 311-20.
16. Ellingson LD, Colbert LH, Cook DB. Physical activity is related to pain sensitivity in healthy women. *Med Sci Sports Exerc* 2012; **44**(7): 1401-6.
17. Landmark T, Romundstad P, Borchgrevink PC, Kaasa S, Dale O. Associations between recreational exercise and chronic pain in the general population: evidence from the HUNT 3 study. *Pain* 2011; **152**(10): 2241-7.
18. World Health Organization. World report on ageing and health.
19. Chien MY, Chen HC. Poor sleep quality is independently associated with physical disability in older adults. *J Clin Sleep Med* 2015; **11**(3): 225-32.
20. Benedict C, Byberg L, Cedernaes J, et al. Self-reported sleep disturbance is associated with Alzheimer's disease risk in men. *Alzheimers Dement* 2015; **11**(9): 1090-7.

For peer review only - http://bmjopen.bmj.com/site/about/guidelines.xhtml

21. Nagai M, Hoshide S, Kario K. Sleep duration as a risk factor for cardiovascular disease- a review of the recent literature. *Curr Cardiol Rev* 2010; **6**(1): 54-61.
22. Cappuccio FP, D'Elia L, Strazzullo P, Miller MA. Quantity and quality of sleep and incidence of type 2 diabetes: a systematic review and meta-analysis. *Diabetes Care* 2010; **33**(2): 414-20.
23. Cappuccio FP, Cooper D, D'Elia L, Strazzullo P, Miller MA. Sleep duration predicts cardiovascular outcomes: a systematic review and meta-analysis of prospective studies. *Eur Heart J* 2011; **32**(12): 1484-92.
24. Johar H, Kawan R, Emeny RT, Ladwig KH. Impaired Sleep Predicts Cognitive Decline in Old People: Findings from the Prospective KORA Age Study. *Sleep* 2016; **39**(1): 217-26.
25. Edwards RR, Almeida DM, Klick B, Haythornthwaite JA, Smith MT. Duration of sleep contributes to next-day pain report in the general population. *Pain* 2008; **137**(1): 202-7.
26. Tiede W, Magerl W, Baumgärtner U, Durrer B, Ehlert U, Treede RD. Sleep restriction attenuates amplitudes and attentional modulation of pain-related evoked potentials, but augments pain ratings in healthy volunteers. *Pain* 2010; **148**(1): 36-42.
27. Roehrs TA, Harris E, Randall S, Roth T. Pain sensitivity and recovery from mild chronic sleep loss. *Sleep* 2012; **35**(12): 1667-72.
28. Dunietz GL, Swanson LM, Jansen EC, et al. Key insomnia symptoms and incident pain in older adults: direct and mediated pathways through depression and anxiety. *Sleep* 2018; **41**(9).
29. Kline CE. The bidirectional relationship between exercise and sleep: Implications for exercise adherence and sleep improvement. *Am J Lifestyle Med* 2014; **8**(6): 375-9.
30. Lowe H, Haddock G, Mulligan LD, et al. Does exercise improve sleep for adults with insomnia? A systematic review with quality appraisal. *Clin Psychol Rev* 2019; **68**: 1-12.
31. Mesas AE, Hagen EW, Peppard PE. The bidirectional association between physical activity and sleep in middle-aged and older adults: a prospective study based on polysomnography. *Sleep* 2018; **41**(9).
32. Li J, Vitiello MV, Gooneratne NS. Sleep in Normal Aging. *Sleep Med Clin* 2018; **13**(1): 1-11.
33. Hirshkowitz M, Whiton K, Albert SM, et al. National Sleep Foundation's sleep time duration recommendations: methodology and results summary. *Sleep Health* 2015; **1**(1): 40-3.
34. Fisher GG, Ryan LH. Overview of the Health and Retirement Study and Introduction to the Special Issue. *Work Aging Retire* 2018; **4**(1): 1-9.
35. Smith D, Wilkie R, Croft P, McBeth J. Pain and Mortality in Older Adults: The Influence of Pain Phenotype. *Arthritis Care Res (Hoboken)* 2018; **70**(2): 236-43.
36. Sparling PB, Howard BJ, Dunstan DW, Owen N. Recommendations for physical activity in older adults. *BMJ* 2015; **350**: h100.
37. He XZ, Baker DW. Differences in leisure-time, household, and work-related physical activity by race, ethnicity, and education. *J Gen Intern Med* 2005; **20**(3): 259-66.
38. Tucker-Seeley RD, Subramanian SV, Li Y, Sorensen G. Neighborhood safety, socioeconomic status, and physical activity in older adults. *Am J Prev Med* 2009; **37**(3): 207-13.
39. Jenkins CD, Stanton BA, Niemcryk SJ, Rose RM. A scale for the estimation of sleep problems in clinical research. *J Clin Epidemiol* 1988; **41**(4): 313-21.
40. West B, Berglund P, Heeringa S. A closer examination of subpopulation analysis of complex-sample survey data. *Stata Journal* 2008; **8**(4): 520-31.
41. Whibley D, AlKandari N, Kristensen K, et al. Sleep and Pain: A Systematic Review of Studies of Mediation. *Clin J Pain* 2019.
42. Baglioni C, Regen W, Teghen A, et al. Sleep changes in the disorder of insomnia: a meta-analysis of polysomnographic studies. *Sleep medicine reviews* 2014; **18**(3): 195-213.

For peer review only - http://bmjopen.bmj.com/site/about/guidelines.xhtml

For peer review only

43. Dijk DJ. Slow-wave sleep deficiency and enhancement: implications for insomnia and its management. *World J Biol Psychiatry* 2010; **11 Suppl 1**: 22-8.
44. Krueger JM, Majde JA, Rector DM. Cytokines in immune function and sleep regulation. *Handb Clin Neurol* 2011; **98**: 229-40.
45. Besedovsky L, Lange T, Born J. Sleep and immune function. *Pflugers Arch* 2012; **463**(1): 121-37.
46. Halson SL. Sleep in elite athletes and nutritional interventions to enhance sleep. *Sports Med* 2014; **44 Suppl 1**: S13-23.
47. Reilly T, Edwards B. Altered sleep-wake cycles and physical performance in athletes. *Physiol Behav* 2007; **90**(2-3): 274-84.
48. Samuels C. Sleep, recovery, and performance: the new frontier in high-performance athletics. *Phys Med Rehabil Clin N Am* 2009; **20**(1): 149-59, ix.
49. Skarpsno ES, Mork PJ, Nilsen TIL, Jørgensen MB, Holtermann A. The joint association of musculoskeletal pain and domains of physical activity with sleep problems: cross-sectional data from the DPhacto study, Denmark. *Int Arch Occup Environ Health* 2019; **92**(4): 491-9.
50. Sluiter JK, Frings-Dresen MH, Meijman TF, van der Beek AJ. Reactivity and recovery from different types of work measured by catecholamines and cortisol: a systematic literature overview. *Occup Environ Med* 2000; **57**(5): 298-315.
51. Cassidy JD, Côté P, Carroll LJ, Kristman V. Incidence and course of low back pain episodes in the general population. *Spine (Phila Pa 1976)* 2005; **30**(24): 2817-23.
52. Dionne CE, Dunn KM, Croft PR. Does back pain prevalence really decrease with increasing age? A systematic review. *Age Ageing* 2006; **35**(3): 229-34.
53. Stamatakis E, Koster A, Hamer M, et al. Emerging collaborative research platforms for the next generation of physical activity, sleep and exercise medicine guidelines: the Prospective Physical Activity, Sitting, and Sleep consortium (ProPASS). *Br J Sports Med* 2019.

For peer review only

For peer review only - http://bmjopen.bmj.com/site/about/guidelines.xhtml

Figure 1. The moderating effect of sleep disturbance on the association between physical activity and later incident pain

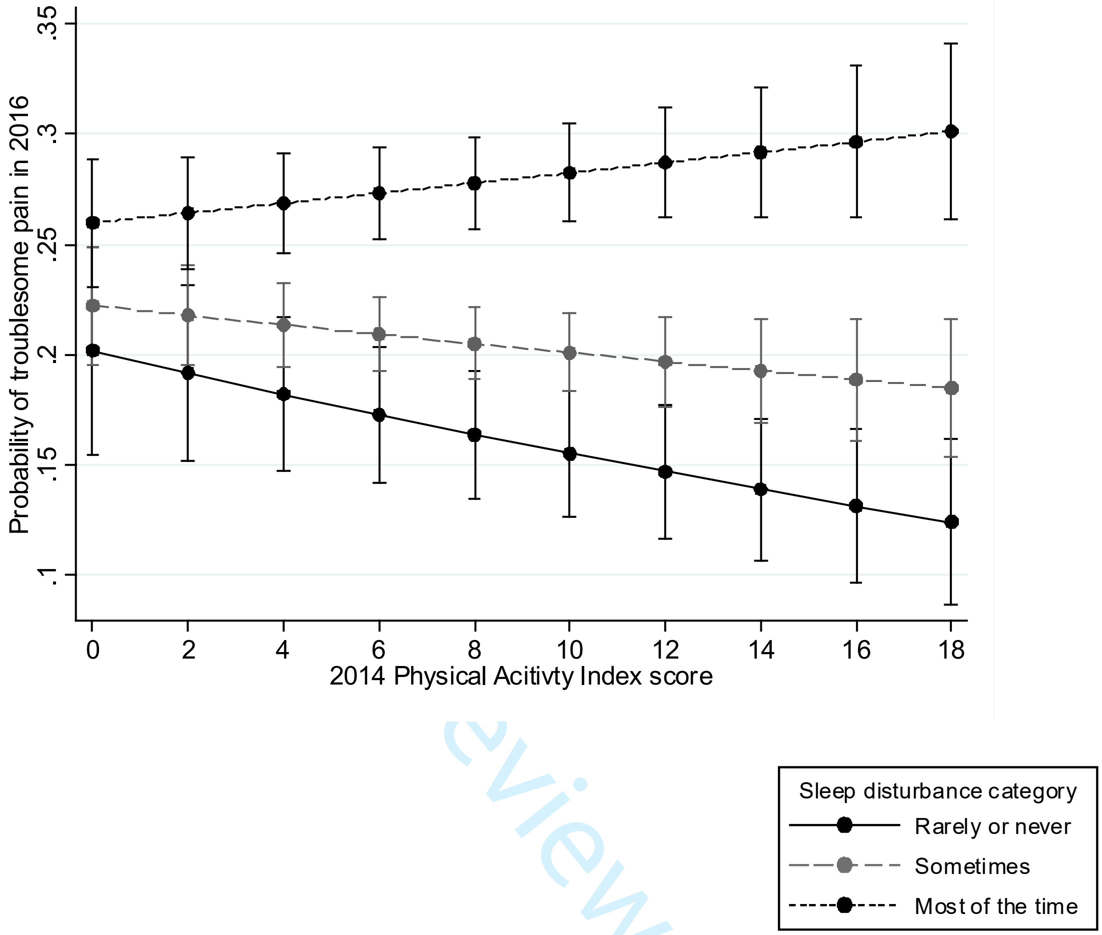

For peer review only

Supplement 1.

Table S1A. Logistic regression analysis investigating the moderating effect of having trouble falling asleep on the association between 2014 physical activity index score and likelihood of troublesome pain in 2016*

	Odds Ratio (95% CI)	Linearized SE	T	P
**Physical activity index score** (PAI)	0.98 (0.97–0.99)	0.007	-2.75	0.008
**Trouble falling asleep**				
Rarely or never	Reference			
Sometimes	1.15 (0.88–1.51)	0.15	1.07	0.29
Most of the time	1.26 (0.91–1.74)	0.20	1.41	0.16
**Physical activity index score * sleep disturbance category interaction term**				
Rarely or never	Reference			
Sometimes	1.02 (1.00–1.05)	0.01	1.88	0.07[a]
Most of the time	1.04 (1.00–1.07)	0.02	2.18	**0.03**[a]
**Age**	0.99 (0.98–1.00)	0.004	-1.54	0.13
**Gender**				
Male	Reference			
Female	0.97 (0.81–1.17)	0.09	-0.31	0.76
**BMI**				
Underweight/normal weight	Reference			
Overweight	1.05 (0.87–1.26)	0.09	0.50	0.62
Obese	1.36 (1.10–1.69)	0.15	2.84	0.006
Obese, BMI ≥35	1.55 (1.21–2.00)	0.19	3.53	0.001
**Race/ethnicity**				
White	Reference			
Black	0.98 (0.81–1.17)	0.09	-0.26	0.79
Hispanic	1.27 (0.92–1.75)	0.21	1.47	0.15
Other	1.23 (0.83–1.82)	0.24	1.07	0.29
**Years of school**	0.95 (0.93–0.97)	0.01	-4.73	<0.001
**History of depression**	1.69 (1.39–2.07)	0.17	5.31	<0.001
**History of major disease**[†]	1.24 (1.03–1.51)	0.12	2.28	0.03
**Arthritis**	2.39 (2.02–2.82)	0.20	10.51	<0.001
**Diabetes**	1.07 (0.93–1.22)	0.07	0.94	0.35

* Adjusted analysis N=8,037
CI: confidence interval; SE: standard error
[a] Wald test statistic for overall interaction: p=0.06
[†] Major disease defined as having a history of cancer (excluding skin), lung disease, heart condition, or stroke.

For peer review only

Table S1B. Logistic regression analysis investigating the moderating effect of having trouble waking up during the night on the association between 2014 physical activity index score and likelihood of troublesome pain in 2016*

	Odds Ratio (95% CI)	Linearized SE	T	P
**Physical activity index score** (PAI)	0.98 (0.96–0.99)	0.008	-2.61	0.01
**Trouble waking up during the night**				
Rarely or never	Reference			
Sometimes	1.08 (0.81–1.43)	0.15	0.51	0.61
Most of the time	1.36 (1.04–1.79)	0.19	2.29	0.03
**Physical activity index score * sleep disturbance category interaction term**				
Rarely or never	Reference			
Sometimes	1.01 (0.99–1.04)	0.01	1.24	0.22[a]
Most of the time	1.03 (1.00–1.06)	0.01	1.92	0.06[a]
**Age**	0.99 (0.98–1.00)	0.004	-1.79	0.08
**Gender**				
Male	Reference			
Female	1.02 (0.85–1.22)	0.09	0.19	0.85
**BMI**				
Underweight/normal weight	Reference			
Overweight	1.06 (0.88–1.27)	0.09	0.65	0.52
Obese	1.36 (1.09–1.70)	0.15	2.81	0.007
Obese, BMI ≥35	1.56 (1.22–1.99)	0.19	3.62	0.001
**Race/ethnicity**				
White	Reference			
Black	1.05 (0.88–1.26)	0.10	0.54	0.59
Hispanic	1.34 (0.97–1.87)	0.22	1.80	0.08
Other	1.32 (0.90–1.95)	0.26	1.43	0.16
**Years of school**	0.94 (0.92–0.96)	0.01	-5.58	<0.001
**History of depression**	1.74 (1.43–2.14)	0.18	5.49	<0.001
**History of major disease**[†]	1.25 (1.03–1.52)	0.12	2.34	0.02
**Arthritis**	2.36 (2.00–2.78)	0.19	10.40	<0.001
**Diabetes**	1.05 (0.91–1.20)	0.07	0.66	0.51

* Adjusted analysis N=8,018
CI: confidence interval; SE: standard error
[a] Wald test statistic for overall interaction: p=0.15
[†] Major disease defined as having a history of cancer (excluding skin), lung disease, heart condition, or stroke.

For peer review only

Table S1c. Logistic regression analysis investigating the moderating effect of having trouble with waking up too early on the association between 2014 physical activity index score and likelihood of troublesome pain in 2016*

	Odds Ratio (95% CI)	Linearized SE	T	P
**Physical activity index score** (PAI)	0.99 (0.98–1.00)	0.007	-1.30	0.20
**Trouble with waking up too early**				
Rarely or never	Reference			
Sometimes	1.26 (1.00–1.59)	0.15	1.98	0.05
Most of the time	1.79 (1.21–2.64)	0.35	3.00	0.004
**Physical activity index score * sleep disturbance category interaction term**				
Rarely or never	Reference			
Sometimes	1.00 (0.98–1.02)	0.01	0.26	0.79[a]
Most of the time	0.99 (0.96–1.03)	0.02	-0.46	0.65[a]
**Age**	0.99 (0.99–1.00)	0.004	-1.48	0.14
**Gender**				
Male	Reference			
Female	1.02 (0.85–1.23)	0.09	0.25	0.80
**BMI**				
Underweight/normal weight	Reference			
Overweight	1.05 (0.88–1.26)	0.10	0.57	0.57
Obese	1.37 (1.10–1.69)	0.15	2.93	0.005
Obese, BMI ≥35	1.55 (1.21–1.98)	0.19	3.55	0.001
**Race/ethnicity**				
White	Reference			
Black	1.00 (0.84–1.20)	0.09	0.04	0.97
Hispanic	1.27 (0.92–1.76)	0.21	1.47	0.15
Other	1.25 (0.85–1.84)	0.24	1.15	0.26
**Years of school**	0.95 (0.93–0.97)	0.01	-4.84	<0.001
**History of depression**	1.77 (1.45–2.16)	0.18	5.67	<0.001
**History of major disease**[†]	1.24 (1.03–1.50)	0.12	2.27	0.03
**Arthritis**	2.38 (2.02–2.81)	0.20	10.58	<0.001
**Diabetes**	1.07 (0.93–1.22)	0.07	0.98	0.33

* Adjusted analysis N=8,034
CI: confidence interval; SE: standard error
[a] Wald test statistic for overall interaction: p=0.83
[†] Major disease defined as having a history of cancer (excluding skin), lung disease, heart condition, or stroke.

For peer review only

Table S1D. Logistic regression analysis investigating the moderating effect of feeling unrested on waking on the association between 2014 physical activity index score and likelihood of troublesome pain in 2016*

	**Odds Ratio (95% CI)**	**Linearized SE**	**T**	**P**
**Physical activity index score** (PAI)	0.99 (0.98–1.00)	0.007	-1.70	0.10
**Feeling unrested on waking**				
Rarely or never	Reference			
Sometimes	1.36 (1.08–1.71)	0.16	2.67	0.01
Most of the time	1.46 (1.10–1.93)	0.21	2.69	0.009
**Physical activity index score * sleep disturbance category interaction term**				
Rarely or never	Reference			
Sometimes	1.00 (0.98–1.03)	0.01	0.12	0.90[a]
Most of the time	1.04 (1.00–1.07)	0.02	1.90	0.06[a]
**Age**	1.00 (0.99–1.00)	0.004	-0.80	0.43
**Gender**				
Male	Reference			
Female	1.03 (0.86–1.23)	0.09	0.37	0.72
**BMI**				
Underweight/normal weight	Reference			
Overweight	1.05 (0.88–1.26)	0.09	0.58	0.57
Obese	1.36 (1.10–1.69)	0.15	2.88	0.006
Obese, BMI ≥35	1.55 (1.20–1.99)	0.19	3.49	0.001
**Race/ethnicity**				
White	Reference			
Black	0.98 (0.82–1.18)	0.09	-0.18	0.86
Hispanic	1.22 (0.88–1.69)	0.20	1.21	0.23
Other	1.27 (0.87–1.87)	0.25	1.25	0.22
**Years of school**	0.95 (0.93–0.97)	0.01	-4.96	<0.001
**History of depression**	1.69 (1.37–2.09)	0.18	4.98	<0.001
**History of major disease**†	1.24 (1.02–1.51)	0.12	2.21	0.03
**Arthritis**	2.37 (2.02–2.79)	0.19	10.65	<0.001
**Diabetes**	1.06 (0.93–1.21)	0.07	0.86	0.39

* Adjusted analysis N=8,041
CI: confidence interval; SE: standard error
[a] Wald test statistic for overall interaction: p=0.17
† Major disease defined as having a history of cancer (excluding skin), lung disease, heart condition, or stroke.

For peer review only

Figure S1. The moderating effect of sleep disturbance domains on the association between physical activity and later incident pain

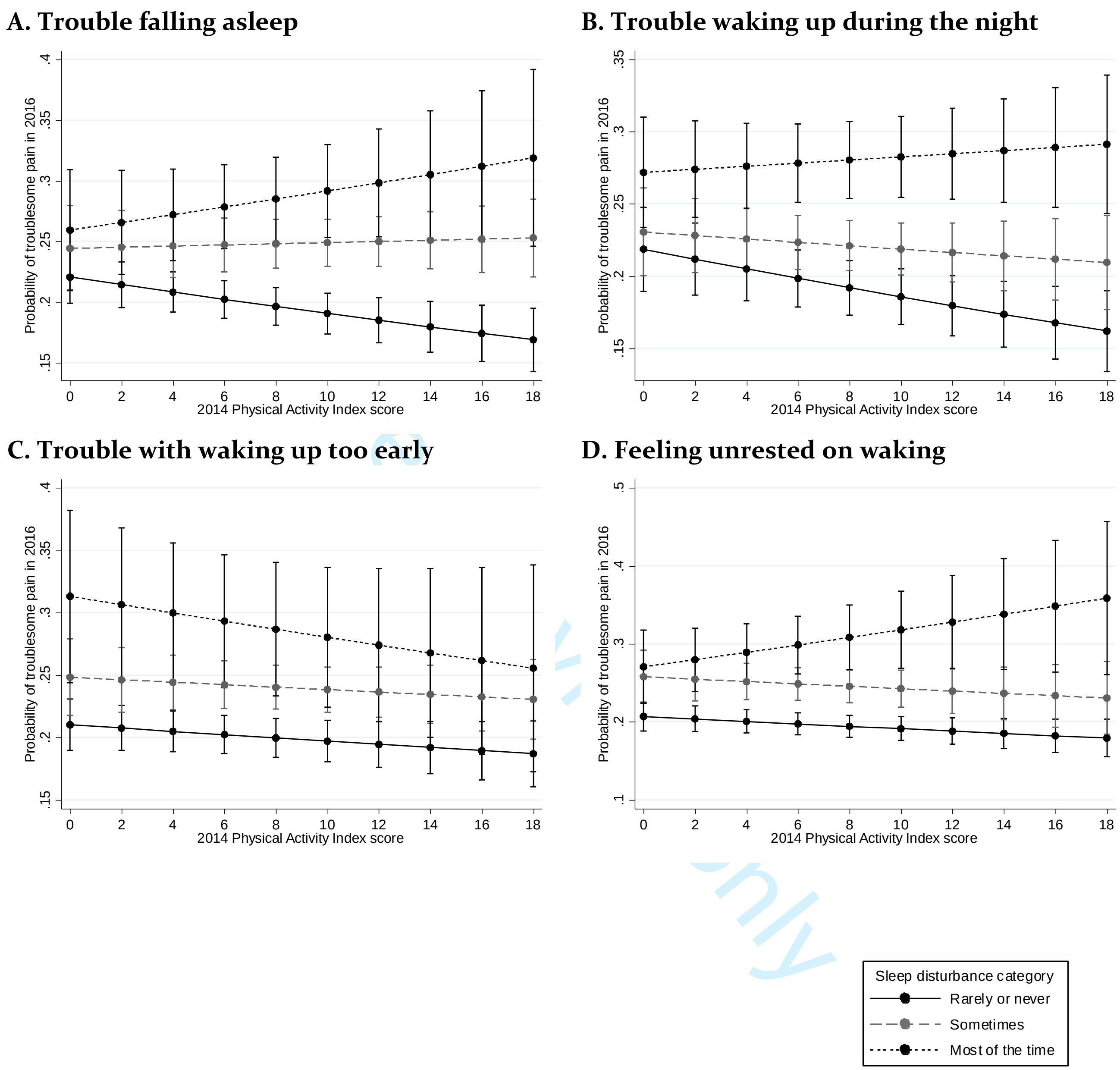

Supplement 2. Logistic regression analysis investigating the moderating effect of sleep disturbance category on the association between 2014 physical activity index score and likelihood of troublesome pain in 2016*, with additional adjustment for living arrangement status**

	OR (95% CI)	SE	T	P
**Physical activity index score** (PAI)	0.97 (0.94–0.99)	0.01	-2.44	0.02
**Sleep disturbance category**				
Rarely or never	Reference			
Sometimes	1.14 (0.78–1.67)	0.22	0.69	0.50
Most of the time	1.43 (0.99–2.06)	0.26	1.95	0.06
**Physical activity index score * sleep disturbance category interaction term**				
Rarely or never	Reference			
Sometimes	1.02 (0.99–1.06)	0.02	1.17	0.25[a]
Most of the time	1.05 (1.01–1.08)	0.02	2.77	0.01[a]
**Age**	0.99 (0.99–1.00)	0.004	-1.47	0.15
**Gender**				
Male	Reference			
Female	1.02 (0.84–1.25)	0.10	0.23	0.82
**BMI**				
Underweight/normal weight	Reference			
Overweight	1.05 (0.88–1.26)	0.09	0.60	0.55
Obese	1.36 (1.09–1.70)	0.15	2.74	0.01
Obese, BMI ≥35	1.57 (1.23–2.01)	0.19	3.66	0.001
**Race/ethnicity**				
White	Reference			
Black	1.02 (0.84–1.23)	0.10	0.18	0.86
Hispanic	1.26 (0.91–1.75)	0.21	1.40	0.17
Other	1.27 (0.86–1.89)	0.25	1.23	0.22
**Years of school**	0.95 (0.93–0.97)	0.01	-5.15	<0.001
**History of depression**	1.69 (1.38–2.06)	0.17	5.19	<0.001
**History of major disease**†	1.24 (1.02–1.51)	0.12	2.19	0.03
**Arthritis**	2.35 (1.99–2.77)	0.19	10.38	<0.001
**Diabetes**	1.05 (0.91–1.20)	0.07	0.71	0.48
**Living arrangement status****				
Married or partnered, living with partner	Reference			
Married or partnered, living with partner	1.22 (0.80–1.86)	0.26	0.94	0.35
Not married or partnered, living with unrelated adult	1.13 (0.50–2.58)	0.47	0.31	0.76
Not married or partnered, living with relative	0.99 (0.76–1.29)	0.13	-0.07	0.95
Not married or partnered, living alone	0.97 (0.79–1.19)	0.10	-0.32	0.75
Unknown	1.06 (0.24–4.75)	0.79	0.08	0.94

* Adjusted analysis N=8,036
OR: Odds Ratio; CI: confidence interval; SE: linearized standard error
[a] Wald test statistic for overall interaction: p=0.02
† Major disease defined as having a history of cancer (excluding skin), lung disease, heart condition, or stroke.
**Nursing home status omitted due to collinearity with living arrangement status. Of the total baseline sample, N= 16,361 were interviewed in the community and N=23 were interviewed in a nursing home in 2014. Of those interviewed in a nursing home, N=7 were also categorized within living arrangement status as 'married or partnered, living with partner'; N=16 were also categorized as 'not married or partnered, living alone'.

For peer review only

STROBE Statement—Checklist of items that should be included in reports of ***cohort studies***

	Item No	Recommendation	Page No
**Title and abstract**	1	(*a*) Indicate the study's design with a commonly used term in the title or the abstract<br>(*b*) Provide in the abstract an informative and balanced summary of what was done and what was found	2
**Introduction**			
Background/rationale	2	Explain the scientific background and rationale for the investigation being reported	4-5
Objectives	3	State specific objectives, including any prespecified hypotheses	5
**Methods**			
Study design	4	Present key elements of study design early in the paper	5-9
Setting	5	Describe the setting, locations, and relevant dates, including periods of recruitment, exposure, follow-up, and data collection	5-6
Participants	6	(*a*) Give the eligibility criteria, and the sources and methods of selection of participants. Describe methods of follow-up<br>(*b*) For matched studies, give matching criteria and number of exposed and unexposed	5-6
Variables	7	Clearly define all outcomes, exposures, predictors, potential confounders, and effect modifiers. Give diagnostic criteria, if applicable	6-8
Data sources/ measurement	8*	For each variable of interest, give sources of data and details of methods of assessment (measurement). Describe comparability of assessment methods if there is more than one group	5-8
Bias	9	Describe any efforts to address potential sources of bias	8-9
Study size	10	Explain how the study size was arrived at	9
Quantitative variables	11	Explain how quantitative variables were handled in the analyses. If applicable, describe which groupings were chosen and why	6-9
Statistical methods	12	(*a*) Describe all statistical methods, including those used to control for confounding<br>(*b*) Describe any methods used to examine subgroups and interactions<br>(*c*) Explain how missing data were addressed<br>(*d*) If applicable, explain how loss to follow-up was addressed<br>(*e*) Describe any sensitivity analyses	8-9
**Results**			
Participants	13*	(a) Report numbers of individuals at each stage of study—eg numbers potentially eligible, examined for eligibility, confirmed eligible, included in the study, completing follow-up, and analysed<br>(b) Give reasons for non-participation at each stage<br>(c) Consider use of a flow diagram	9
Descriptive data	14*	(a) Give characteristics of study participants (eg demographic, clinical, social) and information on exposures and potential confounders<br>(b) Indicate number of participants with missing data for each variable of interest<br>(c) Summarise follow-up time (eg, average and total amount)	9-10
Outcome data	15*	Report numbers of outcome events or summary measures over time	9-10; 13-14

For peer review only - http://bmjopen.bmj.com/site/about/guidelines.xhtml

Main results	16	(*a*) Give unadjusted estimates and, if applicable, confounder-adjusted estimates and their precision (eg, 95% confidence interval). Make clear which confounders were adjusted for and why they were included<br>(*b*) Report category boundaries when continuous variables were categorized<br>(*c*) If relevant, consider translating estimates of relative risk into absolute risk for a meaningful time period	10-11; 13-15
Other analyses	17	Report other analyses done—eg analyses of subgroups and interactions, and sensitivity analyses	10-11; 15; 18; 19
**Discussion**			
Key results	18	Summarise key results with reference to study objectives	16
Limitations	19	Discuss limitations of the study, taking into account sources of potential bias or imprecision. Discuss both direction and magnitude of any potential bias	18-20
Interpretation	20	Give a cautious overall interpretation of results considering objectives, limitations, multiplicity of analyses, results from similar studies, and other relevant evidence	20
Generalisability	21	Discuss the generalisability (external validity) of the study results	18
**Other information**			
Funding	22	Give the source of funding and the role of the funders for the present study and, if applicable, for the original study on which the present article is based	6;20

*Give information separately for exposed and unexposed groups.

For peer review only

For peer review only - http://bmjopen.bmj.com/site/about/guidelines.xhtml
